# Adiposity and mortality among intensive care patients with COVID-19 and non-COVID-19 respiratory conditions: a cross-context comparison study in the UK

**DOI:** 10.1186/s12916-024-03598-3

**Published:** 2024-09-13

**Authors:** Joshua A. Bell, David Carslake, Amanda Hughes, Kate Tilling, James W. Dodd, James C. Doidge, David A. Harrison, Kathryn M. Rowan, George Davey Smith

**Affiliations:** 1 MRC Integrative Epidemiology Unit at the University of Bristol, Bristol, UK; 2 Population Health Sciences, Bristol Medical School, University of Bristol, Bristol, UK; 3 Academic Respiratory Unit, Southmead Hospital, University of Bristol, Bristol, UK; 4 Intensive Care National Audit & Research Centre (ICNARC), London, UK

**Keywords:** Adiposity, BMI, Mortality, COVID-19, Influenza, Pneumonia, Intensive care, Critical care, ICNARC, Epidemiology

## Abstract

**Background:**

Adiposity shows opposing associations with mortality within COVID-19 versus non-COVID-19 respiratory conditions. We assessed the likely causality of adiposity for mortality among intensive care patients with COVID-19 versus non-COVID-19 by examining the consistency of associations across temporal and geographical contexts where biases vary.

**Methods:**

We used data from 297 intensive care units (ICUs) in England, Wales, and Northern Ireland (Intensive Care National Audit and Research Centre Case Mix Programme). We examined associations of body mass index (BMI) with 30-day mortality, overall and by date and region of ICU admission, among patients admitted with COVID-19 (*N* = 34,701; February 2020–August 2021) and non-COVID-19 respiratory conditions (*N* = 25,205; February 2018–August 2019).

**Results:**

Compared with non-COVID-19 patients, COVID-19 patients were younger, less often of a white ethnic group, and more often with extreme obesity. COVID-19 patients had fewer comorbidities but higher mortality. Socio-demographic and comorbidity factors and their associations with BMI and mortality varied more by date than region of ICU admission. Among COVID-19 patients, higher BMI was associated with excess mortality (hazard ratio (HR) per standard deviation (SD) = 1.05; 95% CI = 1.03–1.07). This was evident only for extreme obesity and only during February–April 2020 (HR = 1.52, 95% CI = 1.30–1.77 vs. recommended weight); this weakened thereafter. Among non-COVID-19 patients, higher BMI was associated with lower mortality (HR per SD = 0.83; 95% CI = 0.81–0.86), seen across all overweight/obesity groups and across dates and regions, albeit with a magnitude that varied over time.

**Conclusions:**

Obesity is associated with higher mortality among COVID-19 patients, but lower mortality among non-COVID-19 respiratory patients. These associations appear vulnerable to confounding/selection bias in both patient groups, questioning the existence or stability of causal effects.

**Supplementary Information:**

The online version contains supplementary material available at 10.1186/s12916-024-03598-3.

## Background

The global spread of severe acute respiratory syndrome coronavirus 2 (SARS-CoV-2), and the resultant coronavirus disease 2019 (COVID-19), continues to threaten public health [1]. Identifying modifiable causes of mortality among patients severely ill with COVID-19 remains a priority. In the post-2022 era, this task coincides with the need to manage possible dual surges of severe COVID-19 and influenza-related respiratory diseases, and studies must now consider the impact of risk factors within both conditions to guide appropriate messaging.

Higher adiposity likely causes numerous non-infectious diseases [2] and large-scale evidence, including from Mendelian randomisation (MR) studies, now supports adiposity as a likely cause of SARS-CoV-2 infection and hospitalisation with severe COVID-19, with the available (non-MR) studies suggesting higher adiposity is associated with higher mortality with COVID-19 [3–11]. This contrasts sharply with evidence on patients with non-COVID-19 respiratory conditions, with studies suggesting that higher adiposity, including extreme obesity, is associated with *lower* mortality—although the one available MR study suggests that the causal effect might be positive [12–14]. Nearly all studies estimating the potential effects of adiposity on mortality among patients hospitalised with severe respiratory disease (COVID-19 and non-COVID-19) have been estimated via conventional observational studies which use multivariable adjustments to address confounding and other biases. Such adjustments rarely fully remove bias because they rely on implausible assumptions of no unmeasured confounding and no measurement error—issues which could explain modest effect sizes commonly observed [15, 16]. MR is often unfeasible for assessing mortality in hospital settings given the lack of genetic data at scale in clinically selected samples [5, 7, 8, 10, 11, 14]. As a result, the potential for unmeasured/residual confounding, reverse causation, and selection bias makes causality difficult to infer.

Cross-context comparison is an underutilised tool for causal inference in observational studies. This approach involves directly comparing associations between exposures and outcomes across temporal or geographical contexts where confounding or selection pressures vary. Consistency in the direction and magnitude of exposure-outcome associations across contexts, despite variation in the impact of confounding/selection, builds confidence in the causality of those exposure-outcome associations [17, 18]. Previously, comparisons across geographical contexts where socioeconomic gradients in exposures differ helped to affirm the likely effects of gestational blood glucose on offspring birthweight and of breastfeeding on offspring intelligence, and reduce confidence in the suggested benefits of breastfeeding for offspring adiposity and blood pressure [17, 19]. Cross-context comparisons have not been formally applied to assess the causality of adiposity for mortality among patients severely ill with COVID-19 and non-COVID-19 respiratory conditions, but this is now feasible within the United Kingdom (UK) setting given that the prevalence and impact of confounding/selection factors among patients may differ by time and geography [20–22].

Cross-context comparison requires variation in bias across contexts (time/place), i.e. it can assess the impact of context-varying bias, but not the impact of context-stable bias. Given uniquely rapid changes in COVID-19 management over time and geography, we may expect context-varying bias to influence adiposity-mortality associations more among COVID-19 patients than non-COVID-19 respiratory patients. Moreover, the influence of context-stable bias such as reverse causation, which can be expected to be very stable in situations where external confounders have differing effects, may influence adiposity-mortality associations more among non-COVID-19 respiratory patients. This may be assessable by comparing characteristics of COVID-19 and non-COVID-19 respiratory patients and examining how adiposity-mortality associations differ between them.

The Intensive Care National Audit and Research Centre (ICNARC) has coordinated the collection of data on nearly all patients admitted to intensive care units (ICUs) in England, Wales, and Northern Ireland since 2010 [20, 21, 23, 24]. In this study, we aimed to assess the causality of adiposity for mortality among people hospitalised with severe COVID-19 and non-COVID-19 respiratory conditions using a cross-context comparison approach. We used nationally representative data from the ICNARC Case Mix Programme on patients admitted to ICU with COVID-19 (~ 33,000 patients between February 2020 and August 2021) and with non-COVID-19 respiratory conditions (~ 25,000 patients between February 2018 and August 2019 (pre-pandemic)). Within each patient group, we estimated the overall association between adiposity and mortality. We then examined whether socio-demographic and comorbidity indicators (potential confounding/selection factors) and their associations with adiposity and mortality vary by date and geographical region of ICU admission. Lastly, we examined whether adiposity-mortality associations are consistent across dates and regions with varying confounding/selection pressures.

## Methods

### Study population

**Fig. 1:**
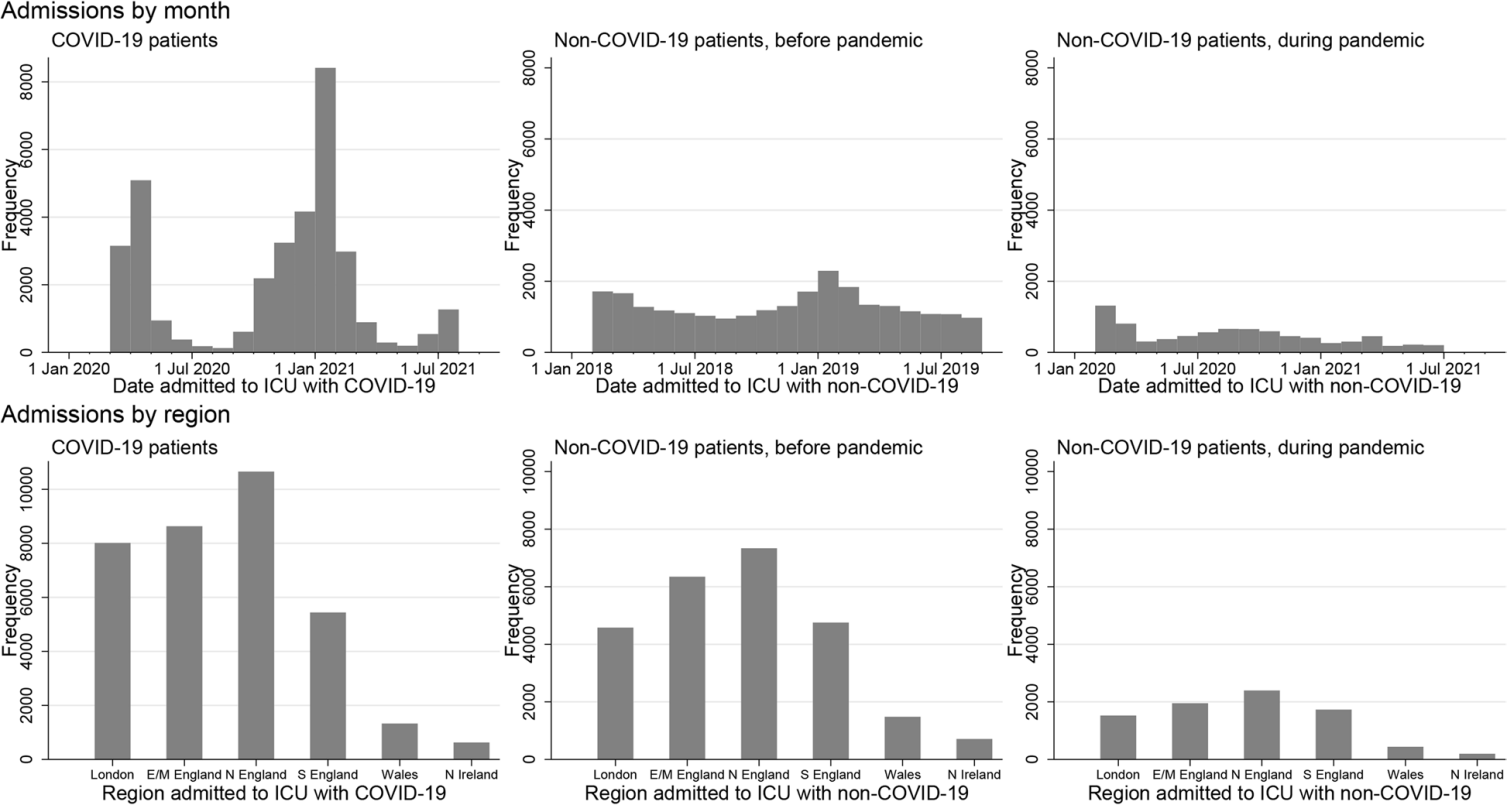
Numbers of patients admitted to any of 280 ICUs in England, Wales, and Northern Ireland with COVID-19 (5 Feb 2020 to 1 Aug 2021) and to any of 266 ICUs with non-COVID-19 respiratory conditions (1 Feb 2018 to 31 Aug 2019 and 1 Feb 2020 to 30 June 2021) participating in the ICNARC Case Mix Programme (297 ICUs overall). E/M East/Midlands, N North, S South. *N* = 34,701 COVID-19 patients, 25,205 non-COVID-19 patients before the pandemic and 8241 non-COVID-19 patients during the pandemic

We included patients aged ≥ 16 years admitted to any of 280 ICUs across England, Wales, and Northern Ireland with COVID-19 confirmed at or after admission between 5 February 2020 and 1 August 2021, plus adult patients admitted to any of 266 ICUs with respiratory diseases which were not COVID-19, including viral and bacterial pneumonia, bronchitis, bronchiolitis, or laryngotracheobronchitis (encompassing suspected/confirmed influenza) between 1 February 2018 and 31 August 2019 (Fig. 1). These start/end dates for COVID-19 admissions were based on data availability when this study commenced; the start/end dates for non-COVID-19 admissions were chosen to match the first/last months of COVID-19 admissions in a similarly long pre-pandemic period. Non-overlapping periods were used for primary analyses because these were expected to involve less disease misclassification and less estimate imprecision for non-COVID-19 respiratory conditions given their relative rarity during COVID-19 waves (e.g. given lockdowns). Data were from ICUs participating in the Case Mix Programme: the national clinical audit covering all National Health Service (NHS) adult, general intensive care, and combined intensive care/high dependency units, plus some additional specialist ICUs and standalone high dependency units, coordinated by ICNARC [23–25]. This audit excludes all paediatric and neonatal ICUs and ICUs in Scotland. The same individual patient could have one COVID-19 record and one or more non-COVID-19 records. Approval for the collection and use of patient-identifiable data without consent in the Case Mix Programme was obtained from the Confidentiality Advisory Group of the Health Research Authority under Sect. 251 of the NHS Act 2006 (approval number PIAG2–10[f]/2005). All data were pseudonymised (patient identifiers removed) prior to extraction for this research.

### Adiposity (exposure)

Body mass index (BMI, as kg/m^2^) was calculated from height and weight which were recorded or estimated by clinicians on admission to ICU. Patient severity and clinician workload often make directly measuring height and weight in the ICU infeasible [26]. About half of the height and weight recordings were visually estimated by clinical staff rather than directly measured for both COVID-19 and non-COVID-19 respiratory patients (53% of COVID-19 patients and 54% of non-COVID-19 respiratory patients had at least one of the height and weight estimated, and 38% of COVID-19 patients and 37% of non-COVID-19 respiratory patients had both height and weight estimated). Previous analyses of ICNARC ICU data supported similar associations of BMI with mortality based on measured vs. estimated values of height and weight [27], and we therefore expected little impact of this measurement error in BMI on BMI-mortality associations aside from potential bias towards the null. We first examined BMI as a continuous variable (per standard deviation (SD) based on z-scores derived separately within COVID-19 and non-COVID-19 groups using all available data). These z-scores were analysed linearly and as cubic splines with five knots at the recommended percentiles [28]. Secondly, we analysed BMI as a categorical variable based on World Health Organization classifications of underweight (< 18.5 kg/m^2^), recommended weight (18.5 to < 25 kg/m^2^), overweight (25.0 to < 30 kg/m^2^), obesity class 1 (30.0 to < 35 kg/m^2^), obesity class 2 (35.0 to < 40 kg/m^2^), and obesity class 3 + (≥ 40.0 kg/m^2^).

### Thirty-day all-cause mortality (outcome)

We included deaths in ICU from any cause (reported directly to ICNARC by hospital staff). Within any continuous hospital stay, a patient’s time in ICU was considered to run from their first ICU admission until their last ICU discharge. We censored analysis time at 30 days after (first) admission to ICU and assumed that patients discharged from ICU (for the last time within a hospital stay) lived to be censored at 30 days post-admission [20]. This was because censoring discharged patients at discharge would have selectively removed the healthiest patients from follow-up (‘informative’ censoring). On the other hand, discharged patients who were subsequently re-admitted to ICU within the same hospital stay were treated as if they had remained in ICU because assuming their survival to 30 days from the first admission would have included many who in fact died by this time. Deaths occurring more than 30 days post-admission were excluded due to the generally high short-term mortality rate within ICU settings; later deaths are rare and risk introducing unrelated causes of death, while the assumption of survival in discharged patients also becomes less plausible with time [23]. Patients with unknown outcomes were assumed to have remained alive in the ICU at the end of their follow-up and were censored at the end of their follow-up (or at 30 days if their ICU stay already exceeded this). Follow-up which ended on the date of admission was considered to have lasted 0.5 days.

### Socio-demographic and comorbidity indicators (confounding and selection factors)

Measured confounders (hypothesised to cause adiposity and mortality) included patient age, sex, ethnic group, socioeconomic deprivation, and the geographical region and date of ICU admission. Age was used as a continuous variable with a potentially non-linear effect modelled with cubic splines with five knots at the recommended percentiles [28]. Ethnic groups included ‘White’, ‘Black’, ‘Asian’ (specifically South Asian), and ‘mixed/other’ (including East Asian), based on 2011 census categories used for NHS data entry. Deprivation was based on quintiles of the Index of Multiple Deprivation (IMD) which summarises several area-level deprivation indicators including income, education, and employment for neighbourhood-level areas within each UK country, derived from patients’ postcodes. Geographic region was grouped as (1) London; (2) East England and Midlands; (3) North-East England, North-West England, and Yorkshire; (4) South-East England and South-West England; (5) Wales; and (6) Northern Ireland. Admission date was considered as six periods for COVID-19 patients: (1) 5 February–30 April 2020; (2) 1 May–31 July 2020; (3) 1 August–31 October 2020; (4) 1 November–31 January 2021; (5) 1 February–30 April 2021; and (6) 1 May–1 August 2021; and six corresponding periods 2 years earlier for non-COVID-19 respiratory patients: (1) 1 February–30 April 2018; (2) 1 May–31 July 2018; (3) 1 August–31 October 2018; (4) 1 November 2018–31 January 2019; (5) 1 February–30 April 2019; and (6) 1 May–31 August 2019.

Several comorbidity indicators were recorded and considered here as selection factors. These are factors thought likely to influence selection into the study (through admission to ICU with COVID-19 or non-COVID-19 respiratory disease) and which may also influence mortality. If adiposity also affects the probability of selection, an induced association between adiposity and the selection factor will bias the estimated effect of adiposity on mortality. However, these selection factors are very plausibly caused by adiposity (whereas adjusted-for covariates are either causal of adiposity or share a common cause with it). Adjustment for selection factors would cause bias by blocking a mediated effect of adiposity on mortality. Additionally, if the selection factor has other causes which also influence mortality, adjustment for the selection factor may induce an association between adiposity and these other causes, further biasing estimates, a mechanism referred to as collider bias [22, 29]. Rather than adjusting for these selection factors, we therefore only measured their associations with adiposity and mortality, as an indication of the potential for bias in the sample. The comorbidity variables examined as selection factors included severe comorbid diseases documented in the patient notes as being present within the 6 months prior to ICU admission: respiratory disease (shortness of breath with light activity or home ventilation), cardiovascular disease (symptoms at rest), end-stage renal disease requiring chronic renal replacement therapy, liver disease (biopsy-proven cirrhosis, portal hypertension or hepatic encephalopathy), metastatic disease, haematological disease (acute or chronic leukaemia, multiple myeloma, or lymphoma) and an immunocompromised state (chemotherapy, radiotherapy, daily high-dose steroids, AIDS, or acquired immunohumoral or cellular immune deficiency). As indicators of acute severity within ICU, we included (1) the acute physiology and chronic health evaluation-II (APACHE-II) score and the ICNARC extreme physiology score which summarise patient physiology within the first 24 h in ICU (higher values being adverse), (2) the ratio of arterial oxygen partial pressure (PaO_2_) to fractional inspired oxygen (FiO_2_) (kPa units) calculated from arterial blood gas with the lowest PaO_2_ in the first 24 h, and (3) the number of days on which advanced respiratory support was received (including ventilation). Additionally, we examined two indicators of pre-ICU health status: whether the patient was physically dependent on others for activities of daily living prior to admission (some dependency; total dependency; or independent), and whether the patient had any past severe illness (defined as having a zero value of the APACHE-II past medical history weighting, grouped as yes/no).

### Statistical approach

#### Analyses informing cross-context comparisons

We described the socio-demographic and comorbidity indicators (confounding/selection factors) of patients admitted to ICU with COVID-19 and non-COVID-19 respiratory conditions via proportions or means and standard deviations. Patient groups were first described overall, and then with separate stratification by time period and region of ICU admission to examine temporal/geographic trends in patient characteristics. These temporal trend descriptions excluded COVID-19 patients admitted during February 2020 and August 2021 due to low case counts (5 and 18 cases, respectively); these patients were not excluded from subsequent regression models because those models use aggregated cases/months only.

We examined associations of socio-demographic and comorbidity indicators (independent variables in separate models) with BMI using linear regression models, and with mortality using Gompertz proportional hazards models, each adjusted for age (cubic splines) and sex. Time since ICU admission was used as the time axis in the survival analyses. The patient group (COVID-19 vs. non-COVID-19) was interacted with all terms allowing separate estimates for each group and a test for heterogeneity between the estimates (using Stata’s post-estimation ‘test’ command). Gompertz models were used as a parametric alternative to semi-parametric Cox models to relax the unrealistic assumption of identical baseline hazards in each patient group and a Gompertz distribution was preferred to the more commonly used Weibull distribution because it gave a closer fit to the observed hazard and survivor functions. These models were then additionally stratified by ICU admission period or region, separately, each in 6 strata as described above. Interactions and independent baseline hazards were used such that separate estimates were made in each combination of stratum and patient condition. Heterogeneity tests were carried out within each patient group, comparing estimates in the six strata.

#### Analyses for overall adiposity-mortality associations and cross-context comparisons

We used Gompertz parametric survival models to estimate the overall association of BMI with 30-day all-cause mortality among ICU patients with COVID-19 and non-COVID-19 respiratory conditions. BMI was first modelled linearly in SD units, then categorically relative to recommended weight and finally, for plotting, as cubic splines of the *z*-scores with five knots at the recommended percentiles [28]. We adjusted for age (cubic splines), sex, ethnic group (4 categories), deprivation (quintile categories), admission period (6 categories), and admission region (6 categories). The time axis, entry, and censoring for these Gompertz models were as described above. The proportional hazards assumption was tested for the linear Gompertz models by splitting follow-up time at the median time to death (10 days for COVID-19 patients and 4 days for non-COVID-19 patients) and comparing hazard ratios (HRs) between these 2 periods. As sensitivity analyses for non-COVID-19 respiratory patients we (1) additionally excluded patients admitted to ICU with bacterial pneumonia (thus considering only viral respiratory conditions for comparison with COVID-19) and (2) considered non-COVID-19 respiratory patients who were admitted during the same months as COVID-19 patients (1 February 2020 to 30 June 2021, since data on non-COVID-19 respiratory patients in July 2021 were not available). In two further sensitivity analyses, we repeated the analyses described above including only those patients (i) with measured (not estimated) height and weight or (ii) who were not dependent on others for the activities of daily living prior to admission.

We then repeated Gompertz models of BMI with mortality among COVID-19 and non-COVID-19 respiratory patients with the same confounding adjustments but with separate stratification by admission date and region, using the groupings noted above. The stratifying variable was omitted from the adjustment set in each analysis. Interactions and independent baseline hazards were used to make estimates equivalent to those from completely separate models in each stratum. Stata’s post-estimation ‘test’ command was used to test for interaction between BMI and each stratifying variable in relation to mortality (smaller *P*-values indicate stronger evidence that associations vary by date/region). Analyses were conducted using Stata 17 and code is available at https://github.com/Carslake/ICU_BMI_Covid/.

## Results

### Characteristics of ICU patients with COVID-19 and non-COVID-19 respiratory conditions

**Fig. 2:**
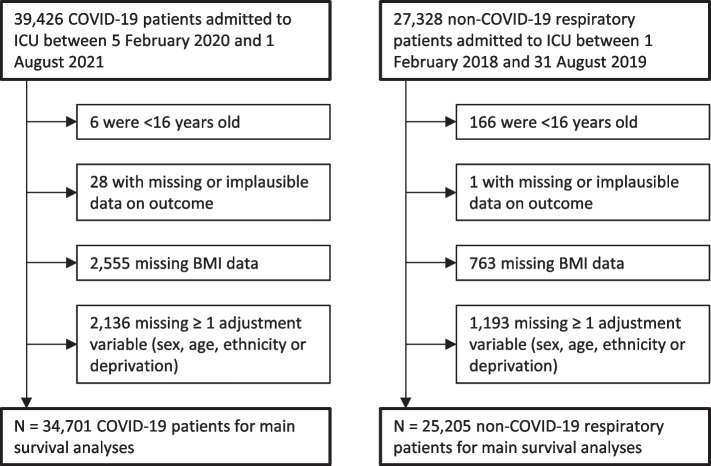
Flow of patients admitted to ICU with COVID-19 and non-COVID-19 respiratory conditions. ICU Intensive care unit, BMI body mass index

Of 39,426 adult patients admitted to ICU with COVID-19 between 5 February 2020 and 1 August 2021, 34,701 (88%) were eligible for the main analyses based on having data on BMI (exposure), sociodemographic adjustment variables, and 30-day ICU mortality (outcome) (Fig. 2). Of 27,328 adult patients admitted to ICU with non-COVID-19 respiratory conditions between 1 February 2018 and 31 August 2019, 25,205 (92%) were eligible for the main analyses based on the same criteria. For both patient groups, data were most often missing for BMI or ethnicity. For the descriptive analysis of comorbidities, patients were further excluded if they were missing the variable in question; this excluded up to 7% of COVID-19 patients and 6% of non-COVID-19 patients.

Compared with non-COVID-19 respiratory patients who were admitted to ICU before the COVID-19 pandemic (the main comparison group for analyses), COVID-19 patients were younger, more often male, more deprived and substantially less often of a white ethnic group (Table 1). COVID-19 patients had a more than twofold higher proportion of Black, and a threefold higher proportion of Asian and mixed, ethnic groups. All of these sociodemographic variables were stable over time among non-COVID-19 patients (Additional files 1–2: Figs. S1–S2), but there were clear temporal differences among COVID-19 patients. The average age declined, particularly after December 2020 (when UK vaccination programmes began). The proportion of males also declined. Deprivation fluctuated considerably but rose overall. The ethnic composition of COVID-19 patients fluctuated, particularly the relative proportions of white and Asian patients, without showing a clear overall trend. Regional variation in age and sex was minimal in both patient groups, but regions differed in levels of deprivation and ethnic composition (Additional files 3–5: Figs. S3–S5). Regional patterns in deprivation were similar for COVID-19 and non-COVID-19 patients, but regional differences in ethnic composition (non-white ethnic groups being most common in London and least common in Northern Ireland) were more pronounced among COVID-19 patients.

**Table 1:** Characteristics of ICU patients in the study sample

Pandemic	**COVID-19 patients**^**a**^	**Non-COVID-19 patients, before pandemic**^**b**^	**Non-COVID-19 patients, during pandemic**^**c**^
	*N* = 32,265 to 34,701	*N* = 23,687 to 25,205	*N* = 7640 to 8241
***Socio-demographics***			
Male sex^d^	66.5% (23,067)	57.2% (14,414)	59.6% (4910)
Age (years)^e^	58.5 (13.4)	62.0 (16.2)	60.7 (15.7)
Age group			
16 to < 35 years^d^	5.8% (2015)	7.2% (1806)	7.6% (627)
35 to < 50 years^d^	17.6% (6091)	14.3% (3608)	15.2% (1249)
50 to < 60 years^d^	25.9% (8972)	17.5% (4407)	19.7% (1627)
60 to < 70 years^d^	29.1% (10,109)	23.2% (5837)	22.9% (1891)
70 to < 80 years^d^	18.5% (6407)	25.2% (6362)	25.5% (2100)
≥ 80 years	3.2% (1107)	12.6% (3185)	9.1% (747)
Ethnic group			
Asian^d^	15.9% (5511)	4.7% (1190)	5.1% (421)
Black^d^	6.8% (2356)	2.8% (704)	3.4% (281)
White^d^	70.1% (24,329)	90.0% (22,682)	88.1% (7264)
Mixed/other^d^	7.2% (2505)	2.5% (629)	3.3% (275)
Index of Multiple Deprivation			
1st quintile (least deprived)^d^	12.7% (4417)	14.9% (3759)	14.1% (1160)
2nd quintile^d^	15.3% (5326)	17.2% (4341)	16.9% (1391)
3rd quintile^d^	18.5% (6408)	19.6% (4932)	19.8% (1635)
4th quintile^d^	24.1% (8353)	22.1% (5577)	22.5% (1852)
5th quintile (most deprived)^d^	29.4% (10,197)	26.2% (6596)	26.7% (2203)
Geographical region			
London, England^d^	23.1% (8014)	18.2% (4577)	18.5% (1527)
East England^d^	9.1% (3175)	10.4% (2615)	10.3% (848)
Midlands, England^d^	15.7% (5457)	14.8% (3731)	13.4% (1105)
North-east and Yorkshire, England^d^	14.1% (4879)	16.5% (4165)	16.9% (1395)
North-west England^d^	16.7% (5778)	12.6% (3169)	12.1% (1000)
South-east England^d^	10.9% (3794)	11.9% (3006)	12.9% (1065)
South-west England^d^	4.7% (1648)	6.9% (1749)	8.1% (667)
Wales^d^	3.8% (1328)	5.9% (1481)	5.3% (438)
Northern Ireland^d^	1.8% (628)	2.8% (712)	2.4% (196)
***Current adiposity***			
BMI (kg/m^2^)^e^	30.9 (7.7)	27.6 (7.6)	28.2 (8.1)
BMI category			
Underweight (< 18.5 kg/m^2^)^d^	0.8% (280)	5.3% (1334)	4.8% (398)
Recommended (18.5– < 25 kg/m^2^)^d^	20.7% (7181)	37.0% (9323)	34.6% (2855)
Overweight (25– < 30 kg/m^2^)^d^	31.9% (11,069)	29.7% (7475)	29.5% (2430)
Obesity 1 (30– < 35 kg/m^2^)^d^	23.5% (8142)	14.9% (3763)	15.8% (1303)
Obesity 2 (35– < 40 kg/m^2^)^d^	12.2% (4237)	6.9% (1743)	7.8% (645)
Obesity 3 + (≥ 40 kg/m^2^)^d^	10.9% (3792)	6.2% (1567)	7.4% (610)
***Prior or current comorbidities***			
Any past severe illness^d^	9.0% (3126)	21.4% (5398)	20.7% (1710)
Some or total dependency^d^	11.3% (3904)	33.9% (8531)	32.0% (2628)
Very severe cardiovascular disease^d^	0.6% (213)	2.0% (501)	1.5% (125)
Severe respiratory disease^d^	1.0% (340)	6.0% (1499)	5.2% (425)
Liver disease^d^	0.6% (204)	2.1% (528)	2.4% (199)
End-stage renal disease^d^	1.7% (598)	2.1% (533)	2.1% (170)
Metastatic disease^d^	0.6% (208)	2.8% (713)	2.9% (236)
Haematological disease^d^	1.8% (613)	4.3% (1091)	4.5% (372)
Immunocompromised^d^	3.7% (1251)	9.9% (2481)	9.9% (816)
APACHE II acute severity score^e^	14.5 (5.2)	17.9 (6.4)	17.5 (6.4)
ICNARC extreme physiology score^e^	18.5 (7.6)	19.2 (8.6)	19.2 (8.6)
PaO_2_/FiO_2_ ratio^e^	16.3 (9.5)	20.5 (11.0)	20.9 (11.0)
Advanced respiratory support (days)^e^	10.7 (15.8)	4.4 (9.7)	4.5 (9.4)
***Death rate post-ICU admission***			
30-day all-cause mortality^d^	34.3% (11,912)	22.4% (5637)	23.0% (1892)

Patients admitted to intensive care units in England, Wales, and Northern Ireland with COVID-19 and non-COVID-19 respiratory conditions eligible for the main analyses and the sensitivity analysis of non-COVID-19 patients during the pandemic

*ICU* Intensive care unit, *BMI* Body mass index

^a^COVID-19 patients admitted between 5^th^ February 2020 and 1^st^ August 2021

^b^non-COVID-19 respiratory patients admitted between 1^st^ February 2018 and 31^st^ August 2019

^c^non-COVID-19 respiratory patients admitted between 1^st^ February 2020 and 30^th^ June 2021

^d^Categorical variables summarised as percentage (number)

^e^Continuous variables summarised as mean (standard deviation)

COVID-19 patients had a higher mean BMI, at 30.9 kg/m^2^ vs. 27.6 kg/m^2^ in non-COVID-19 patients (Table 1). They presented as underweight one-sixth as often and as recommended weight nearly half as often, yet presented with obesity, particularly classes 2 and 3 + obesity, about twice as often. Mean BMI was relatively stable over time among non-COVID-19 patients but showed a modest increase among COVID-19 patients, starting at 29.8 kg/m^2^ in March 2020 and ending at 31.7 kg/m^2^ in July 2021 (Additional file 2: Fig. S2). This corresponded to a fall in the proportion of recommended weight and overweight patients while the proportion of patients with obesity rose, e.g. from 7.6% in March 2020 to 13.2% in July 2021 for class 3 + obesity (Additional file 6: Fig. S6). There was regional heterogeneity in BMI, with a lower mean in London reflected in corresponding differences across the BMI categories. This heterogeneity was apparent among both patient groups but was more pronounced among COVID-19 patients (Additional file 5: Fig. S5, Additional file 7: Fig. S7). The proportion of BMI which was estimated rather than measured was similar overall for COVID-19 and non-COVID-19 patients but was a little more variable over time in the former group, with the most estimation in spring 2020 and winter 2020–2021, when admissions were highest (Fig. 1 and Additional file 8: Fig. S8). Regional variation was similar in COVID-19 and non-COVID-19 patients, with weight most frequently estimated in Northern Ireland (Additional file 9: Fig. S9).

COVID-19 patients had less prior severe illness than non-COVID-19 patients (9.0% vs. 21.4%) and less pre-ICU dependency on others for activities of daily living (11.3% vs. 33.9%; Table 1). They also presented less often with severe comorbidities including cardiovascular, respiratory, liver, renal, metastatic, haematological, respiratory, and immunocompromising diseases, compared to non-COVID-19 patients. These pre-existing conditions showed limited variation over time and between regions, to a similar extent for COVID-19 and non-COVID-19 patients (Additional files 6 and 10: Figs. S6 and S10). COVID-19 patients had a lower PaO_2_/FiO_2_ ratio and more days of advanced respiratory support, indicating worse respiratory function than non-COVID-19 patients, but lower overall severity of illness as indicated by mean APACHE-II and ICNARC scores (Table 1). While these severity indicators remained relatively constant over time among non-COVID-19 patients, they all declined over time among COVID-19 patients (indicating less severe illness except for the PaO_2_/FiO_2_ ratio which suggests the opposite; Additional file 8: Fig. S8, Additional file 11: Fig. S11). The ICNARC physiological severity score and the days of advanced respiratory support both showed more variability between regions in COVID-19 patients than among non-COVID-19 patients (Additional files 9 and 12: Figs. S9 and S12). In contrast, the PaO_2_/FiO_2_ ratio was more variable between regions among non-COVID-19 patients.

Despite being younger with fewer comorbidities, COVID-19 patients experienced higher 30-day ICU mortality at 34.3% vs. 22.4% for non-COVID-19 patients (Table 1). This difference declined substantially over time, as mortality among COVID-19 patients fell from 41.4% in March 2020 to 14.5% in July 2021 (Additional file 8: Fig. S8). In contrast, mortality among non-COVID-19 respiratory patients was relatively stable over time, at 23.9% in March 2018 and 21.0% in July 2019. Regional variation in mortality was similar for both conditions, being highest in Wales (39.8% of COVID-19 patients and 27.5% of non-COVID-19 patients; Additional file 13: Fig. S13).

The characteristics of non-COVID-19 patients admitted to ICU during the COVID-19 pandemic (a smaller comparison group for sensitivity analyses) were not substantially different from those of non-COVID-19 patients admitted pre-pandemic (Table 1). They were a little more similar to COVID-19 patients, e.g. younger age, higher proportions of non-white ethnic groups, higher mean BMI, and less historical illness, indicating a greater potential for misclassification with COVID-19 in this overlapping admissions period.

### Associations of confounding/selection factors with BMI and mortality among ICU patients with COVID-19 and non-COVID-19 respiratory conditions

Among COVID-19 patients, non-white ethnic groups were associated with lower BMI, particularly the Asian group at − 2.77 kg/m^2^ (95% confidence interval (CI) = − 2.99, − 2.56) (Additional file 14: Table S1). There was considerable temporal and regional heterogeneity in this association, e.g. Asian ethnic group was most associated with lower BMI in South England (excluding London) and Northern Ireland (Additional files 15–16: Tables S2–S3). Among non-COVID-19 patients, an association between BMI and ethnic group was also present but the association, and its temporal and regional heterogeneity, were weaker (Additional files 17–18: Tables S4–S5). While the temporal and regional heterogeneity among COVID-19 patients was strongest for the association between Asian ethnicity and BMI, heterogeneity among non-COVID-19 patients was greatest for the association between Black ethnicity and BMI.

Asian ethnicity was associated with higher mortality in both patient groups but more so in COVID-19 patients (Additional file 14: Table S1). There was no evidence of temporal or regional heterogeneity in this association (Additional files 19–22: Tables S6–S9). Black ethnicity was associated with slightly higher mortality among COVID-19 patients (HR = 1.04, 95% CI = 0.97, 1.12) and lower mortality among non-COVID-19 patients (HR = 0.67, 95% CI = 0.54, 0.82) but the reverse was found among white patients (HR = 0.85, 95% CI = 0.82, 0.89 and HR = 1.12, 95% CI = 1.02, 1.23, respectively). Higher mortality among Black COVID-19 patients was apparent only in the first admissions period (February–April 2020, HR = 1.22, 95% CI = 1.09, 1.37); the association was null or slightly protective during each period thereafter.

Higher deprivation was similarly associated with higher BMI in COVID-19 and non-COVID-19 patients (Additional file 14: Table S1). There was strong evidence of regional heterogeneity in this association among non-COVID-19 patients (Additional file 18: Table S5) and a suggestion of temporal heterogeneity among COVID-19 patients (Additional file 15: Table S2).

Deprivation was associated with higher mortality among COVID-19 patients but not among non-COVID-19 patients. These associations were largely consistent across regions and admission dates (Additional files 14, 19–22: Tables S1, S6–S9).

Most comorbidities were associated with lower BMI in both patient groups, but magnitudes were often higher among COVID-19 patients (Additional file 14: Table S1). In contrast, pre-ICU dependency and comorbid respiratory disease (the definition of which includes home ventilation due to obesity-related sleep disorders) were associated with higher BMI in both groups. Heterogeneity tests indicated that the magnitude of many of these associations between BMI and comorbidities varied over time among COVID-19 patients (Additional file 15: Table S2) but there was much less evidence of temporal heterogeneity among non-COVID-19 patients (Additional file 17: Table S4). There was little evidence of regional heterogeneity in associations between BMI and comorbidities in either patient group (Additional files 16 and 18: Tables S3 and S5).

Comorbidities were almost always associated with higher mortality (Additional file 14: Table S1). The magnitude of these associations often differed between COVID-19 and non-COVID-19 patients, being usually stronger in non-COVID-19 patients. An interesting exception was renal disease, which was consistently associated with higher mortality among COVID-19 patients but lower mortality among non-COVID-19 patients. Other associations between comorbidities and mortality showed considerable temporal heterogeneity among COVID-19 patients, tending to increase over the study period (Additional file 19: Table S6) but there was little temporal heterogeneity in the comorbidity-mortality association among non-COVID-19 patients (Additional file 21: Table S8). There was some evidence of regional heterogeneity in the associations between comorbidities and mortality, particularly among non-COVID-19 patients (Additional files 20 and 22: Tables S7 and S9).

### Overall associations of BMI with mortality among ICU patients with COVID-19 and non-COVID-19 respiratory conditions

**Fig. 3:**
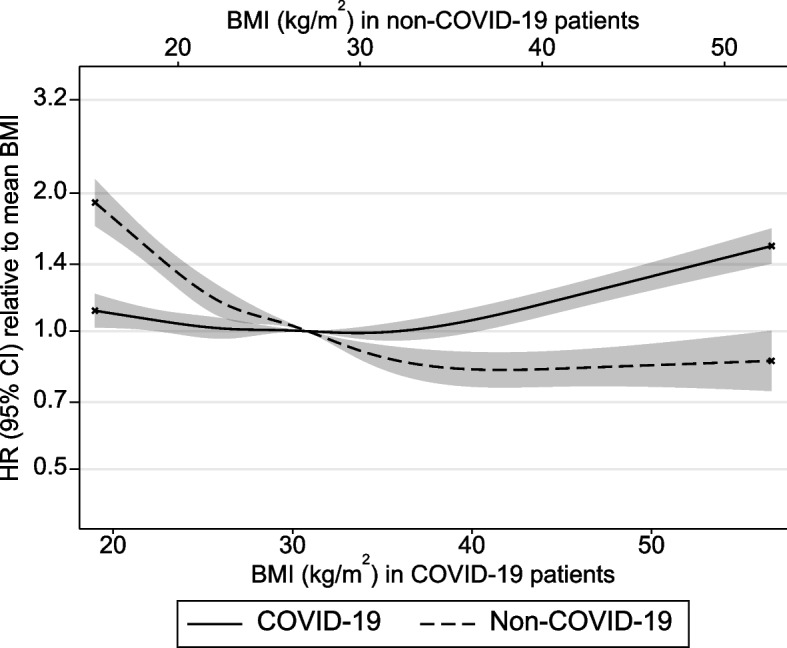
Association between BMI and mortality in COVID-19 and non-COVID-19 respiratory patients. BMI body mass index, HR hazard ratio, CI confidence interval. Results from parametric survival analyses with Gompertz baseline hazard functions. BMI was modelled as a cubic spline with five knots and converted back from condition-specific standard deviations to kg/m^2^ for display. Survival time was censored at 30 days with patients discharged earlier assumed to survive to 30 days. Adjusted for sex, age (cubic spline), ethnic group, deprivation, admission period and admission region. Crosses indicate the truncation of plots at the 1st and 99th percentiles of condition-specific BMI *z*-scores

Among COVID-19 patients admitted to ICU, higher BMI (per SD, or 7.6 kg/m^2^) was associated with a small excess ICU mortality (HR = 1.05; 95% CI = 1.03, 1.07; Table 2). Compared with recommended weight patients, underweight was associated with excess mortality at HR = 1.27 (95% CI = 1.04, 1.55), whereas there was little evidence that overweight, class 1 obesity, or class 2 obesity were associated with ICU mortality. Mortality was elevated with class 3 + obesity vs. recommended weight at HR = 1.22 (95% CI = 1.14, 1.32). Cubic spline models (Fig. 3) also indicated that the positive overall association between BMI and mortality derived mainly from those with a BMI over 35–40 kg/m^2^.

**Table 2:** Overall associations of BMI with 30-day all-cause mortality among ICU patients with respiratory conditions

	**Hazard ratio (95% confidence interval) for 30-day all-cause mortality**			
	**COVID-19 patients**^**a**^	**Non-COVID-19 patients, before pandemic**^**b**^	**Non-COVID-19 patients, during pandemic**^**c**^	**Non-COVID-19 patients, excluding bacterial pneumonia**^**d**^
Deaths (N)	11,912 (34,701)	5637 (25,205)	1892 (8,241)	4452 (19,535)
Per SD higher BMI	1.05 (1.03, 1.07)	0.83 (0.81, 0.86)	0.90 (0.86, 0.95)	0.84 (0.81, 0.87)
Underweight (< 18.5 kg/m^2^)	1.27 (1.04, 1.55)	1.52 (1.36, 1.69)	1.41 (1.15, 1.72)	1.53 (1.35, 1.72)
Recommended (18.5– < 25 kg/m^2^)	1.00 (reference)	1.00 (reference)	1.00 (reference)	1.00 (reference)
Overweight (25– < 30 kg/m^2^)	1.00 (0.95, 1.05)	0.84 (0.79, 0.89)	0.86 (0.77, 0.96)	0.85 (0.79, 0.91)
Obesity 1 (30– < 35 kg/m^2^)	0.96 (0.91, 1.01)	0.73 (0.67, 0.79)	0.76 (0.66, 0.88)	0.74 (0.68, 0.82)
Obesity 2 (35– < 40 kg/m^2^)	0.96 (0.90, 1.03)	0.72 (0.64, 0.81)	0.74 (0.61, 0.91)	0.74 (0.64, 0.84)
Obesity 3 + (≥ 40 kg/m^2^)	1.22 (1.14, 1.32)	0.72 (0.63, 0.82)	0.89 (0.73, 1.09)	0.69 (0.60, 0.81)

Results from parametric survival analyses with Gompertz baseline hazard functions. Survival time was censored at 30 days with patients discharged earlier assumed to survive to 30 days. Adjusted for sex, age (cubic spline), ethnic group, deprivation, admission period and admission region. The number of deaths and sample size within each BMI category is reported in Table S12

*BMI* Body mass index, *ICU* Intensive care unit, *SD* Standard deviation

^a^COVID-19 patients admitted between 5th February 2020 and 1st August 2021

^b^Non-COVID-19 respiratory patients admitted between 1st February 2018 and 31st August 2019

^c^Non-COVID-19 respiratory patients admitted between 1st February 2020 and 30th June 2021

^d^Non-COVID-19 respiratory patients, excluding bacterial pneumonia, admitted between 1st February 2018 and 31st August 2019

Among non-COVID-19 patients admitted to ICU, higher BMI (per SD, or 7.5 kg/m^2^) was associated with lower ICU mortality (HR = 0.83; 95% CI = 0.81, 0.86). Compared with recommended weight patients, those who were underweight had substantial excess mortality at HR = 1.52 (95% CI = 1.36, 1.69), whereas patients who were overweight or with obesity had substantially lower mortality (e.g. HR = 0.72; 95% CI = 0.63, 0.82 for class 3 + obesity). The cubic spline models suggested progressively lower mortality with BMI up to BMI in excess of 35–40 kg/m^2^, above which mortality remained consistently low.

The pattern and magnitude of these associations were similar when (i) considering non-COVID-19 patients admitted during the same months as COVID-19 patients (Table 2), (ii) excluding non-COVID-19 patients admitted to ICU with bacterial pneumonia (thus considering only viral respiratory conditions; Table 2), (iii) considering only those patients with measured BMI (Additional file 23: Table S10) or excluding patients who were dependent on others for the activities of daily living prior to admission (Additional file 24: Table S11). The first and third of these suggested weakly that mortality in non-COVID-19 patients with class 3 + obesity might not be reduced to the degree suggested by the main analysis (but was still reduced relative to recommended weight). However, these estimates were less precise owing to smaller sample size and non-COVID-19 patients admitted during the pandemic may have been more prone to disease misclassification.

Proportional hazards tests splitting follow-up at the median time to death suggested that both the positive BMI-mortality association in COVID-19 patients and the negative one in non-COVID-19 patients attenuated towards the null with increasing time spent in ICU. This was rather more pronounced in non-COVID-19 patients (HR = 0.76, 95% CI = 0.72, 0.80 in the first 4 days in ICU; HR = 0.90, 95% CI = 0.86, 0.94 subsequently; *P*_difference_ < 0.0001) than in COVID-19 patients (HR = 1.07, 95% CI = 1.04, 1.10 in the first 10 days in ICU; HR = 1.03, 95% CI = 1.00, 1.06 subsequently; *P*_difference_ = 0.07). In the presence of non-proportional hazards, average HR can be sensitive to the censoring distribution [30, 31]. In our main analyses, 13.3% of COVID-19 patients and 4.9% of non-COVID-19 patients were administratively censored at 30 days, with 7.6% and 4.0% of all recorded deaths, respectively, occurring after this censoring. Censoring before 30 days was very rare (1.2% of COVID-19 patients and 0.5% of non-COVID-19 patients). Importantly for our interest in heterogeneity, both forms of censoring were similar across regions and dates of admission, except for COVID-19 patients in the final period (May–August 2021), when there was less administrative censoring (4.4%) and more censoring before 30 days (20.2%).

### Temporal cross-context comparison: associations of BMI with mortality among ICU patients with COVID-19 and non-COVID-19 respiratory conditions, by date of ICU admission

Among COVID-19 patients admitted to ICU, higher BMI (per SD) was associated with higher mortality (HR = 1.11, 95% CI = 1.06, 1.15) during February–April 2020 (Table 3); this association weakened in May–July 2020 and thereafter with a small positive association during the most populous period of November 2020–January 2021 (HR = 1.04, 95% CI = 1.01, 1.07; BMI-date interaction *P* = 0.015). Compared with recommended weight patients, mortality was highest for patients with class 3 + obesity in February–April 2020 (HR = 1.52, 95% CI = 1.30, 1.77), reducing thereafter. A small positive gradient in risk associated with overweight and classes 1 and 2 obesity (vs. recommended weight) was seen only during February–April 2020, with HRs for these BMI groups (versus recommended weight) < 1 or null across subsequent periods. Being underweight was associated with higher mortality in each period except May–July 2020, but estimates were imprecise given the rarity of underweight COVID-19 patients. The cubic spline plots of continuous BMI (Additional file 25: Fig. S14) support the patterns observed for categories of BMI and mortality among COVID-19 patients.

**Table 3:** Temporal cross-context comparison: associations of BMI with 30-day all-cause mortality among ICU patients with COVID-19 and non-COVID-19 respiratory conditions, by admission date

	**Hazard ratio (95% confidence interval) for 30-day all-cause mortality**						***P***_**interaction**_^**a**^
	**Feb–Apr**	**May–Jul**	**Aug–Oct**	**Nov–Jan**	**Feb–Apr**	**May–Aug**	
Deaths_COVID-19_ (*N*_COVID-19_)	3173 (8,248)	431 (1506)	993 (2928)	5673 (15,828)	1313 (4163)	329 (2028)	
Deaths_Non-COVID-19_ (*N*_Non-COVID-19_)	1071 (4,654)	750 (3313)	683 (3169)	1182 (5305)	997 (4480)	954 (4284)	
**COVID-19 patients admitted 5th February 2020 to 1st August 2021**							
Per SD higher BMI	1.11 (1.06, 1.15)	0.93 (0.83, 1.04)	1.06 (0.98, 1.15)	1.04 (1.01, 1.07)	1.01 (0.95, 1.07)	1.06 (0.95, 1.19)	0.015
Underweight (< 18.5 kg/m^2^)	1.30 (0.82, 2.06)	0.94 (0.49, 1.82)	1.05 (0.51, 2.12)	1.26 (0.90, 1.78)	1.22 (0.75, 1.98)	2.05 (1.06, 3.97)	
Recommended (18.5– < 25 kg/m^2^)	1.00 (reference)	1.00 (reference)	1.00 (reference)	1.00 (reference)	1.00 (reference)	1.00 (reference)	
Overweight (25– < 30 kg/m^2^)	1.06 (0.97, 1.16)	0.91 (0.72, 1.16)	1.09 (0.92, 1.29)	0.99 (0.92, 1.07)	0.88 (0.75, 1.02)	0.84 (0.61, 1.15)	
Obesity 1 (30– < 35 kg/m^2^)	1.04 (0.93, 1.15)	0.85 (0.64, 1.13)	0.93 (0.76, 1.13)	0.96 (0.88, 1.04)	0.85 (0.72, 1.00)	0.80 (0.57, 1.12)	
Obesity 2 (35– < 40 kg/m^2^)	1.13 (0.98, 1.30)	0.77 (0.53, 1.12)	1.06 (0.84, 1.34)	0.94 (0.85, 1.04)	0.72 (0.59, 0.88)	1.12 (0.76, 1.65)	
Obesity 3 + (≥ 40 kg/m^2^)	1.52 (1.30, 1.77)	0.84 (0.56, 1.27)	1.18 (0.91, 1.55)	1.17 (1.06, 1.30)	1.05 (0.85, 1.29)	1.11 (0.72, 1.71)	
**Non-COVID-19 patients admitted 1st February 2018 to 31st August 2019**							
Per SD higher BMI	0.81 (0.75, 0.87)	0.86 (0.78, 0.93)	0.77 (0.70, 0.85)	0.93 (0.88, 0.99)	0.83 (0.77, 0.90)	0.75 (0.70, 0.82)	0.001
Underweight (< 18.5 kg/m^2^)	1.60 (1.26, 2.04)	1.70 (1.27, 2.27)	1.46 (1.07, 1.99)	1.48 (1.17, 1.88)	1.25 (0.95, 1.64)	1.69 (1.33, 2.14)	
Recommended (18.5– < 25 kg/m^2^)	1.00 (reference)	1.00 (reference)	1.00 (reference)	1.00 (reference)	1.00 (reference)	1.00 (reference)	
Overweight (25– < 30 kg/m^2^)	0.82 (0.71, 0.95)	0.86 (0.73, 1.02)	0.72 (0.60, 0.86)	0.95 (0.82, 1.09)	0.85 (0.73, 0.99)	0.77 (0.66, 0.90)	
Obesity 1 (30– < 35 kg/m^2^)	0.76 (0.63, 0.92)	0.62 (0.48, 0.80)	0.66 (0.51, 0.84)	0.85 (0.71, 1.02)	0.71 (0.58, 0.87)	0.69 (0.57, 0.85)	
Obesity 2 (35– < 40 kg/m^2^)	0.61 (0.45, 0.82)	0.76 (0.55, 1.07)	0.82 (0.59, 1.15)	0.84 (0.66, 1.07)	0.76 (0.58, 1.01)	0.54 (0.40, 0.75)	
Obesity 3 + (≥ 40 kg/m^2^)	0.60 (0.43, 0.85)	1.02 (0.73, 1.42)	0.57 (0.38, 0.85)	0.97 (0.75, 1.28)	0.59 (0.42, 0.83)	0.62 (0.45, 0.86)	

Results from parametric survival analyses with Gompertz baseline hazard functions. Survival time was censored at 30 days with patients discharged earlier assumed to survive to 30 days. Adjusted for sex, age (cubic spline), ethnic group, deprivation and admission region. The number of deaths and sample size within each BMI category and period of admission is reported in Table S13

*BMI* Body mass index, *ICU* Intensive care unit

^a^*P*-value from an interaction between admission period and BMI

Among non-COVID-19 respiratory patients admitted to ICU, higher BMI (per SD) was associated with lower mortality across admission dates, e.g. HR = 0.81 (95% CI = 0.75, 0.87) in February–April 2018, but with varying strength of association (BMI-date interaction *P* = 0.001, indicating stronger evidence of heterogeneity in association magnitude than for COVID-19). Mortality was highest among underweight patients across all periods, e.g. HR = 1.60 (95% CI = 1.26, 2.04) during February–April 2018. In the same period, mortality was lower with overweight and each obesity group vs. recommended weight, e.g. HR = 0.60 (95% CI = 0.43, 0.85) for class 3 + obesity. This lower mortality with class 3 + obesity was seen in most periods except for May–July 2018 and November 2018–January 2019 when mortality did not differ from recommended weight. The cubic spline plots of continuous BMI reiterate both the variability and the imprecision of the BMI-mortality association among non-COVID-19 patients with BMI above 40 kg/m^2^.

### Geographical cross-context comparison: associations of BMI with mortality among ICU patients with COVID-19 and non-COVID-19 respiratory conditions, by region of ICU admission

Among COVID-19 patients admitted to ICU, the association of BMI (per SD higher) with higher mortality did not vary across regions (BMI-region interaction *P* = 0.703; Table 4). Compared with recommended weight, mortality was consistently elevated with class 3 + obesity across regions, with HR varying between 1.14 (95% CI = 0.94, 1.38) in southern England and an imprecise 1.29 (95% CI = 0.74, 2.25) in Northern Ireland. Neither overweight nor class 1 or 2 obesity were clearly associated with mortality in any region though there was a strong but imprecisely estimated association with class 2 obesity in Northern Ireland (HR = 1.49, 95% CI = 0.90, 2.46). The cubic spline plots (Additional file 26: Fig. S15) also do not show clear differences between regions in the association between BMI and mortality.

**Table 4:** Geographical cross-context comparison: associations of BMI with 30-day all-cause mortality among ICU patients with COVID-19 and non-COVID-19 respiratory conditions, by admission region

	**Hazard ratio (95% confidence interval) for 30-day all-cause mortality**						***P***_**interaction**_^**a**^
	**London, England**	**E England and Midlands**	**NE and NW England, Yorkshire**	**SE and SW England**	**Wales**	**Northern Ireland**	
Deaths_COVID-19_ (*N*_COVID-19_)	2721 (8014)	3109 (8632)	3690 (10,657)	1669 (5442)	528 (1328)	195 (628)	
Deaths_Non-COVID-19_ (*N*_Non-COVID-19_)	913 (4577)	1461 (6346)	1770 (7334)	954 (4755)	408 (1481)	131 (712)	
**COVID-19 patients admitted 5th February 2020 to 1st August 2021**							
Per SD higher BMI	1.04 (0.99, 1.09)	1.07 (1.02, 1.11)	1.06 (1.03, 1.10)	1.03 (0.97, 1.09)	1.01 (0.91, 1.11)	1.09 (0.95, 1.26)	0.703
Underweight (< 18.5 kg/m^2^)	1.14 (0.79, 1.65)	1.33 (0.90, 1.98)	1.37 (0.95, 1.98)	1.34 (0.71, 2.52)	n/a (n < 5)	1.08 (0.25, 4.65)	
Recommended (18.5– < 25 kg/m^2^)	1.00 (reference)	1.00 (reference)	1.00 (reference)	1.00 (reference)	1.00 (reference)	1.00 (reference)	
Overweight (25– < 30 kg/m^2^)	0.99 (0.90, 1.08)	1.00 (0.90, 1.10)	1.03 (0.94, 1.13)	0.95 (0.83, 1.08)	1.07 (0.84, 1.35)	1.41 (0.92, 2.17)	
Obesity 1 (30– < 35 kg/m^2^)	1.03 (0.92, 1.15)	0.89 (0.80, 1.00)	0.97 (0.88, 1.08)	0.95 (0.83, 1.10)	1.00 (0.77, 1.30)	1.23 (0.78, 1.95)	
Obesity 2 (35– < 40 kg/m^2^)	0.87 (0.74, 1.03)	0.97 (0.85, 1.10)	1.03 (0.91, 1.17)	0.92 (0.77, 1.11)	0.87 (0.63, 1.21)	1.49 (0.90, 2.46)	
Obesity 3 + (≥ 40 kg/m^2^)	1.25 (1.05, 1.48)	1.23 (1.06, 1.42)	1.26 (1.11, 1.44)	1.14 (0.94, 1.38)	1.18 (0.84, 1.66)	1.29 (0.74, 2.25)	
**Non-COVID-19 patients admitted 1st February 2018 to 31st August 2019**							
Per SD higher BMI	0.78 (0.72, 0.85)	0.81 (0.76, 0.87)	0.84 (0.80, 0.89)	0.87 (0.80, 0.94)	0.82 (0.73, 0.93)	0.94 (0.77, 1.14)	0.386
Underweight (< 18.5 kg/m^2^)	1.89 (1.50, 2.38)	1.32 (1.05, 1.65)	1.59 (1.32, 1.91)	1.49 (1.13, 1.95)	0.90 (0.53, 1.52)	1.92 (0.95, 3.89)	
Recommended (18.5– < 25 kg/m^2^)	1.00 (reference)	1.00 (reference)	1.00 (reference)	1.00 (reference)	1.00 (reference)	1.00 (reference)	
Overweight (25– < 30 kg/m^2^)	0.85 (0.72, 0.99)	0.80 (0.71, 0.91)	0.87 (0.77, 0.97)	0.81 (0.69, 0.94)	0.86 (0.69, 1.08)	0.92 (0.59, 1.43)	
Obesity 1 (30– < 35 kg/m^2^)	0.74 (0.59, 0.93)	0.64 (0.54, 0.75)	0.71 (0.61, 0.83)	0.87 (0.71, 1.05)	0.68 (0.49, 0.94)	1.00 (0.60, 1.68)	
Obesity 2 (35– < 40 kg/m^2^)	0.70 (0.50, 0.98)	0.73 (0.59, 0.92)	0.72 (0.58, 0.89)	0.64 (0.47, 0.88)	0.76 (0.49, 1.18)	0.76 (0.36, 1.63)	
Obesity 3 + (≥ 40 kg/m^2^)	0.70 (0.47, 1.02)	0.60 (0.46, 0.78)	0.77 (0.62, 0.96)	0.89 (0.65, 1.21)	0.48 (0.27, 0.87)	1.07 (0.48, 2.43)	

Results from parametric survival analyses with Gompertz baseline hazard functions. Survival time was censored at 30 days with patients discharged earlier assumed to survive to 30 days. Adjusted for sex, age (cubic spline), ethnic group, deprivation and admission period. Results derived from fewer than 5 patients are not shown. The number of deaths and sample size within each BMI category and region of admission is reported in Table S14

*BMI* Body mass Index, *ICU* Intensive care unit

^a^*P*-value from an interaction between admission period and BMI

Among non-COVID-19 patients admitted to ICU, the association of BMI (per SD higher) with lower mortality did not vary across regions (BMI-region interaction *P* = 0.385; Table 4). Compared with recommended weight, underweight was associated with higher mortality in all regions except Wales (HR = 0.90, 95% CI = 0.53, 1.52). The magnitude of these positive associations varied between East England and Midlands (HR = 1.32, 95% CI = 1.05, 1.65) and London (HR = 1.89, 95% CI = 1.50, 2.38) or the less precise Northern Ireland (HR = 1.92, 95% CI = 0.95, 3.89). In most regions, mortality continued to decline across higher BMI groups above recommended weight, with class 3 + obesity often associated with the lowest risk, e.g. at HR = 0.60 (95% CI = 0.46, 0.78) in East England and Midlands and HR = 0.48 (95% CI = 0.27, 0.87) in Wales. The cubic spline plots (Additional file 26: Fig. S15) also suggest regional variation in the degree of elevated mortality at low BMI and show no evidence (albeit with low precision) of elevated mortality at very high BMI, relative to mean BMI.

The numbers of deaths and total sample sizes for each BMI category, admission period, and admission region contributing to the main survival models described above are shown in Additional files 27–29: Tables S12–S14.

## Discussion

We aimed in this study to assess the likely causality of adiposity for mortality among patients severely ill with COVID-19 and non-COVID-19 respiratory conditions using a cross-context comparison approach with nationally representative ICU data in the UK. Consistent adiposity-mortality associations despite varying confounding/selection would increase confidence in causality. Our results suggest that higher adiposity, primarily extreme obesity, is associated with higher mortality among patients admitted to ICU with COVID-19, but lower mortality among patients admitted with non-COVID-19 respiratory conditions both before and during the COVID-19 pandemic. These associations appear vulnerable to confounding/selection bias in both patient groups, questioning the existence or stability of causal effects. Among COVID-19 patients, unfavourable obesity-mortality associations differ substantially by ICU admission date, perhaps reflecting high levels of temporal heterogeneity in potential confounding and selection bias. Among non-COVID-19 respiratory patients, obesity-mortality associations were consistently favourable but varied in magnitude, despite apparently stable circumstances of the measured potential bias. The strong associations of comorbidities with both BMI and mortality (whether stable or unstable) suggest that comorbidity-induced weight loss may bias BMI-mortality associations in both conditions, but particularly among non-COVID-19 patients due to their higher prevalence of comorbidities.

The two contexts examined here were the date and geographical region of ICU admission. These were chosen because the prevalence/level of confounding and selection factors relevant to adiposity-mortality effects differ by time and geography among ICU patients with COVID-19 [25], and thus the effects of those confounders/selectors on adiposity/mortality might also differ among them, although this had not been previously examined. Such context-varying bias is expected to be most influential for COVID-19 given rapid changes in its viral biology and clinical/public management, whereas context-stable bias is expected to be most influential for non-COVID-19 respiratory conditions which was assessed here by comparing characteristics between patient groups. The ability of COVID-19 to cause severe disease in otherwise healthy people, especially prior to vaccination, at a time of restricted social contact also suggests that COVID-19 patients in ICU include a potentially varying proportion of occupationally exposed patients, with a distinct covariate profile. Our results suggest that the associations of confounding/selection factors with BMI and mortality varied mostly by date (not region) of ICU admission, and particularly for COVID-19. For example, among COVID-19 patients, the Black ethnic group was associated with slightly different degrees of mostly lower BMI across admission dates, and with higher mortality only during February–April 2020. This diminishing association with mortality over time has also been seen in the UK general population outside of ICU settings [32]. Deprivation showed little variation in its association with BMI or mortality by date but several comorbidity indicators (e.g. liver disease and APACHE score) had varying associations with BMI and/or mortality.

Notably, higher BMI was not consistently associated with higher mortality across admission dates among COVID-19 patients, with ~ 50% higher mortality for extreme obesity relative to recommended weight seen only within the earliest period of February–April 2020. The negative obesity-mortality associations among non-COVID-19 patients were a little more consistent over time in direction and perhaps magnitude but still displayed heterogeneity well beyond that expected by chance. This consistency among non-COVID-19 patients does not necessarily support causality in that group, however, as this could still reflect stable forms of bias such as reverse causation. This was supported by the high proportion (compared to COVID-19 patients) of non-COVID-19 patients admitted to ICU who were underweight and/or had comorbidities. Such patients might be expected to experience both higher mortality and comorbidity-induced weight loss/cachexia.

Adiposity-mortality associations may differ over time because of changing bias or because of genuine changes in causality, e.g. due to changes in viral variants, immunological naivety, vaccines, and therapeutics. Obesity may have increased COVID-19 mortality mostly during the early pandemic stage (February–April 2020) because this was when all individuals were immunologically naïve to SARS-CoV-2, and excess adiposity may have weakened immune system responses to this new virus; whereas later admission periods will have included more patients who have experienced repeat infections [33–35]. COVID-19 vaccines were introduced in the UK in December 2020 and uptake was earliest among the oldest and most clinically vulnerable (including both extreme obesity and underweight)—the same populations who present most often to ICU. Vaccines substantially reduce mortality from SARS-CoV-2 infection and COVID-19 [36], and along with improved therapeutics likely explain overall declines in mortality, acute severity, and mean age of patients admitted to ICU with COVID-19 post-2020. Notably, however, our results suggest that adiposity-mortality associations among COVID-19 patients started diminishing in May–July 2020, before vaccines were introduced in December 2020. This suggests that vaccines/therapeutics are not the sole reason for variation over time in adiposity-mortality associations among COVID-19 patients and that, in addition to changes in natural immunological naivety of the population, confounding/selection bias, which also varied over time, played a role. For example, collider (selection) bias [22, 29, 37] could have manifested in COVID-19 patients being admitted to ICU at higher BMIs yet with fewer comorbidities over the course of the pandemic, following early evidence on obesity-mortality associations [4, 20]. Our descriptions of patient characteristics over time do suggest increased mean/median BMI of patients admitted to ICU with COVID-19 from July 2020, while the prevalence of past severe illness declined/fluctuated over the same period. These negative BMI-comorbidity associations among COVID-19 patients selected into ICU could have biased obesity-mortality associations in February–April 2020 when obesity was associated with higher mortality, and/or in later time periods where obesity appears unrelated to mortality.

Our results and the results of earlier descriptive reports by ICNARC [25] indicate that obesity, including extreme obesity, is more common within COVID-19 than non-COVID-19 respiratory conditions. It is well known that severe COVID-19 occurs more frequently in patients with more comorbidities in the general population [38], but our results suggest that, within ICU, patients with COVID-19 tend to have fewer comorbidities than patients with non-COVID-19 respiratory conditions. This is despite obesity being more common among COVID-19 patients. This same pattern was also seen in the Netherlands, based on one study using ICU data which compared the adiposity and comorbidity profile of ~ 2600 ICU patients with COVID-19 vs. ~ 2900 with non-COVID-19 viral pneumonia [39]. That Dutch study reported the same contrasting pattern of BMI-mortality associations between patient groups: positive among COVID-19 patients and negative among non-COVID-19 respiratory patients. MR studies of adiposity and COVID-19 mortality do not exist for comparison, including for clinically selected patient samples, except for one MR study which examined ‘critical respiratory illness’ as a composite outcome of death, intubation, or advanced oxygen support, which supported a detrimental effect of BMI [5]. This obesity paradox in ICU is striking and may help reveal the potential for patient selection and reverse causation to bias BMI-mortality associations within severe respiratory disease more broadly—with COVID-19 vs. non-COVID-19 offering another type of cross-context comparison to assess context-stable bias. Pre-existing disease may reduce BMI and raise mortality, and this may explain long-standing observations of higher mortality with underweight and lower mortality with obesity (vs. recommended weight) among respiratory disease patients [12, 13]. Indeed, MR estimates, which should be less prone to confounding by pre-existing disease, suggest that higher adiposity raises pneumonia mortality [14], although MR analyses are lacking for hospitalised patients specifically given the lack of genetic data at scale. Given the relatively healthy comorbidity profile of COVID-19 patients, associations between BMI and mortality among them may be less subject to reverse causation and thus inform on the likely causality of BMI for mortality among both COVID-19 and non-COVID-19 patients (if these conditions are clinically similar). This is important given the need for appropriate clinical messaging around obesity during potential dual burdens of COVID-19 and influenza in future. Our results suggest unfavourable obesity-mortality associations among COVID-19 patients (who have fewer comorbidities), in contrast to favourable obesity-mortality associations among non-COVID-19 patients (who have more comorbidities). If comorbidity-induced weight loss is expected to bias associations, then these results suggest that obesity may not be protective in either group, and that weight loss/maintenance advice applies to both groups.

Over a dozen previous studies examined associations of BMI with mortality within severe COVID-19; all used conventional observational designs and most were small scale (*N* < 500), with larger studies suggesting that excess mortality is driven by extreme obesity, where this was examined [40–45]. Given the global spread of COVID-19, the totality of studies examining adiposity-mortality associations naturally provides a comparison of these associations across temporal and geographical contexts, but the study designs and methods used differ in many other respects and no previous study directly compared dates or regions using the same analytical strategy. One UK study examined how associations between ethnicity and mortality among COVID-19 patients changed over calendar time, but did not examine BMI [32]. Importantly, none of these past studies directly compared the associations of confounding/selection factors with adiposity and mortality across contexts to appraise their impact. Previous studies also tended to statistically adjust for comorbidities in main effect models, which may be an overadjustment and could induce collider bias given that comorbidities can result from adiposity. With cross-context comparisons, however, it is difficult to identify specific confounding or selection factors which underpin any differences in exposure-outcome associations; multiple factors are likely influential, many of which are likely unmeasured and are only proxied by factors which are stratified on. Interpreting results necessarily relies on critical judgement and assessing causality is inherently qualitative.

### Limitations

This study is observational and associations are subject to confounding, selection bias, reverse causation, and measurement error. Cross-context comparisons are intended to interrogate the extent and impact of such biases on exposure-outcome associations but offer incomplete and qualitative assessments. Measurement error may be problematic for adiposity given that this was measured indirectly using BMI which correlates less well with more objective measures of fat mass in severely ill vs. young healthy populations, although the correlation between BMI and abdominal fat area is ~ 0.7 among severely ill adults [46, 47]. The relationship between BMI and percentage body fat may also differ between ethnicity groups, although the extent of this is variable [48, 49]. Data are also collected within ‘real world’ ICU settings and are often recorded less accurately than in research-grade clinics. BMI measures here were a mixture of directly measured and visually estimated values upon ICU admission, and these proportions varied more over time for COVID-19 vs. non-COVID-19 patients (reflecting changing staff workloads/resources during virus waves). The extent of estimated values was similar between patient groups, however, and these different methods of BMI recording have previously shown consistent associations with ICU mortality [27]. Furthermore, the exclusion of estimated BMI values in a sensitivity analysis did not change the overall BMI-mortality associations beyond their confidence limits. The estimation of BMI appeared to be most common when COVID-19 admissions were highest, which probably reflects staff workloads at the time. Staff workload and ICU capacity (including seasonal variation in non-COVID-19 admissions) might cause temporal variation in the potential for bias in BMI-mortality associations if they affect admission criteria and/or clinical practice.

Our study is limited to data which are routinely collected in ICU settings nationally, and thus data on other adiposity measures such as waist circumference and lifestyle factors such as smoking, diet, and physical activity were not available. Smoking history data would be particularly useful for assessing confounding, e.g. where excess mortality with underweight in non-COVID-19 may have been partly confounded by the effects of smoking on weight loss and mortality. Comorbid disease indicators were also limited to very severe forms of disease and excluded less severe diseases which are still relevant to mortality, such as type 2 diabetes and other cardiovascular diseases. One Dutch ICU study did, however, record diabetes history in patients with COVID-19 and non-COVID-19 pneumonia and found no difference between groups (~ 20% in each) [39].

Proportional hazards tests found that both the positive BMI-mortality associations among COVID-19 patients and the negative ones among non-COVID-19 patients attenuated towards the null with increasing time in ICU. This could be because mortality disproportionately removes frailer patients, causing a decline in the hazard over time, but more so at those levels of BMI where mortality is highest [50]. A marginal hazard ratio under non-proportional hazards can be interpreted as an average effect over the support of the data, but its value can be sensitive to the censoring distribution [30, 31]. Had we chosen to administratively censor at more than 30 days, it is therefore likely that HR would have been a little closer to the null, particularly for COVID-19 patients. Because of our interest in heterogeneity between regions or dates of admission, heterogeneity in the censoring distribution is of concern to us. This was only apparent in the final period for COVID-19 patients (May to August 2021), when there was less administrative censoring (i.e. at 30 days) and much more earlier censoring (people who were still in ICU when data collection ended). It is therefore reassuring that results for this period were not particularly different from the others (with the possible exception of the very high HR for underweight people, which should be interpreted with caution). Rather, it is the first period for COVID-19 patients which was distinctive from the others.

Lastly, the ICU data used here for COVID-19 patients were representative of adult ICU patients in England, Wales, and Northern Ireland, but likely excluded individuals who were most extremely clinically vulnerable as they would have been shielding during peak stages of the pandemic and thus presenting less than usual to ICU, whereas equivalently extremely vulnerable non-COVID-19 respiratory patients in 2018–2019 would have presented more readily. Hospital practices were also atypical during COVID-19 surges and many patients severely ill with COVID-19 were likely managed outside of ICU on regular wards due to limited capacity. Such practices would have likely varied more by time than by geography given that national clinical guidance and protocols were rapidly shared across regions of the UK via the NHS during the pandemic. However, BMI-mortality associations among non-COVID-19 patients during the pandemic resembled closely those from 2018 to 2019 and were similarly different from contemporary BMI-mortality associations among COVID-19 patients, suggesting that patient selection affects BMI-mortality associations much less than the reason for admission does. We did not account for potential pseudoreplication due to the same individuals having multiple non-COVID-19 admissions (or both COVID-19 and non-COVID-19 admissions) but this would have been rare in the short study period.

## Conclusions

Our results based on a cross-context comparison approach with nationally representative ICU data in the UK suggest that higher adiposity, primarily extreme obesity, is associated with higher mortality among patients admitted to ICU with COVID-19, but lower mortality among patients admitted with non-COVID-19 respiratory conditions. If these associations among COVID-19 patients had remained consistent despite the observed temporal heterogeneity in potential confounding/selection bias, it would have increased our willingness to interpret them as causal. However, BMI-mortality associations among COVID-19 patients differed by admission date, questioning the existence or stability of causal effects. Among non-COVID-19 respiratory patients, there was less temporal or regional heterogeneity in potential bias, diminishing the power of this approach to test causation. However, the relatively stable and strong associations of comorbidities with both BMI and mortality in this patient group, coupled with their high prevalence of comorbidity, suggest that favourable obesity-mortality associations among non-COVID-19 respiratory patients may reflect comorbidity-induced weight loss.

## Supplementary Information

Additional file 1: Figure S1 Age and sex profiles of ICU patients with COVID-19 (1 March 2020 to 31 July 2021) and non-COVID-19 respiratory conditions (1 Feb 2018 to 31 Aug 2019), by admission date.

Additional file 2: Figure S2 Ethnic group, deprivation and adiposity profiles of ICU patients with COVID-19 (1 March 2020 to 31 July 2021) and non-COVID-19 respiratory conditions (1 Feb 2018 to 31 Aug 2019), by admission date.

Additional file 3: Figure S3 Age profiles of ICU patients with COVID-19 (5 Feb 2020 to 1 Aug 2021) and non-COVID-19 respiratory conditions (1 Feb 2018 to 31 Aug 2019), by geographical region.

Additional file 4: Figure S4 Sex and ethnic group profiles of ICU patients with COVID-19 (5 Feb 2020 to 1 Aug 2021) and non-COVID-19 respiratory conditions (1 Feb 2018 to 31 Aug 2019), by geographical region.

Additional file 5: Figure S5 Deprivation and adiposity profiles of ICU patients with COVID-19 (5 Feb 2020 to 1 Aug 2021) and non-COVID-19 respiratory conditions (1 Feb 2018 to 31 Aug 2019), by geographical region.

Additional file 6: Figure S6 Adiposity, dependency and comorbidity profiles of ICU patients with COVID-19 (1 March 2020 to 31 July 2021) and non-COVID-19 respiratory conditions (1 Feb 2018 to 31 Aug 2019), by admission date.

Additional file 7: Figure S7 Adiposity and prior dependency profiles of ICU patients with COVID-19 (5 Feb 2020 to 1 Aug 2021) and non-COVID-19 respiratory conditions (1 Feb 2018 to 31 Aug 2019), by geographical region.

Additional file 8: Figure S8 Respiratory support, BMI reporting and mortality profiles of ICU patients with COVID-19 (1 March 2020 to 31 July 2021) and non-COVID-19 respiratory conditions (1 Feb 2018 to 31 Aug 2019), by date of admission.

Additional file 9: Figure S9 Respiratory support and BMI reporting profiles of ICU patients with COVID-19 (1 March 2020 to 31 July 2021) and non-COVID-19 respiratory conditions (1 Feb 2018 to 31 Aug 2019), by geographical region.

Additional file 10: Figure S10 Comorbidity and acute severity profiles of ICU patients with COVID-19 (5 Feb 2020 to 1 Aug 2021) or non-COVID-19 respiratory conditions (1 Feb 2018 to 31 Aug 2019), by geographical region.

Additional file 11: Figure S11 Acute severity, physiological severity and respiratory severity profiles of ICU patients with COVID-19 (1 March 2020 to 31 July 2021) and non-COVID-19 respiratory conditions (1 Feb 2018 to 31 Aug 2019), by admission date.

Additional file 12: Figure S12 Physiological and respiratory severity profiles of ICU patients with COVID-19 (5 Feb 2020 to 1 Aug 2021) or non-COVID-19 respiratory conditions (1 Feb 2018 to 31 Aug 2019), by geographical region.

Additional file 13: Figure S13 Mortality profiles of ICU patients with COVID-19 (1 March 2020 to 31 July 2021) and non-COVID-19 respiratory conditions (1 Feb 2018 to 31 Aug 2019), by geographical region.

Additional file 14: Figure S14 Association between BMI and mortality in COVID-19 and non-COVID-19 respiratory patients, by admission date.

Additional file 15: Figure S15 Association between BMI and mortality in COVID-19 and non-COVID-19 respiratory patients, by geographical region.

Additional file 16: Table S1 Associations of confounding and selection factors with BMI and 30-day all-cause mortality among ICU patients with COVID-19 (5 Feb 2020 to 1 Aug 2021) and non-COVID-19 respiratory conditions (1 Feb 2018 to 31 Aug 2019).

Additional file 17: Table S2 Associations of confounding/selection factors with BMI among ICU patients with COVID-19, by admission date

Additional file 18: Table S3 Associations of confounding/selection factors with BMI among ICU patients with COVID-19, by admission region

Additional file 19: Table S4 Associations of confounding/selection factors with BMI among ICU patients with non-COVID-19 respiratory conditions, by admission date

Additional file 20: Table S5 Associations of confounding/selection factors with BMI among ICU patients with non-COVID-19 respiratory conditions, by admission region

Additional file 21: Table S6 Associations of confounding/selection factors with all-cause mortality among ICU patients with COVID-19, by admission date

Additional file 22: Table S7 Associations of confounding/selection factors with all-cause mortality among ICU patients with COVID-19, by admission region

Additional file 23: Table S8 Associations of confounding/selection factors with all-cause mortality among ICU patients with non-COVID-19 respiratory conditions, by admission date

Additional file 24: Table S9 Associations of confounding/selection factors with all-cause mortality among ICU patients with non-COVID-19 respiratory conditions, by admission region

Additional file 25: Supplementary Table S10 Main analyses of all-cause mortality and BMI, restricted to ICU patients whose BMI was measured, not estimated.

Additional file 26: Supplementary Table S11 Main analyses of all-cause mortality and BMI, restricted to ICU patients who were not physically dependent on others for the activities of daily living prior to admission.

Additional file 27: Table S12 Number of deaths and total sample size for each BMI category in Table 2.

Additional file 28: Table S13 Number of deaths and total sample size for each BMI category and admission period in Table 3.

Additional file 29: Table S14 Number of deaths and total sample size for each BMI category and admission region in Table 4.

## Data Availability

Individual-level data are available via application to ICNARC (managed access).

## Abbreviations

APACHE-II: Acute physiology and chronic health evaluation-II
BMI: Body mass index
CI: Confidence interval
COVID-19: Coronavirus disease 2019
FiO_2_: Fractional inspired oxygen
HR: Hazard ratio
ICNARC: Intensive Care National Audit and Research Centre
ICU: Intensive care unit
IMD: Index of Multiple Deprivation
MR: Mendelian randomisation
NHS: National Health Service
PaO_2_: Arterial oxygen partial pressure
SARS-CoV-2: Severe acute respiratory syndrome coronavirus 2
SD: Standard deviation
UK: United Kingdom

## References

[1] Sachs JD, Karim SSA, Aknin L, Allen J, Brosbøl K, Colombo F, et al. The Lancet Commission on lessons for the future from the COVID-19 pandemic. Lancet. 2022;400(10359):1224–80. 36115368 10.1016/S0140-6736(22)01585-9 PMC9539542

[2] Censin JC, Peters SA, Bovijn J, Ferreira T, Pulit SL, Mägi R, et al. Causal relationships between obesity and the leading causes of death in women and men. PLoS Genet. 2019;15(10):e1008405. 31647808 10.1371/journal.pgen.1008405 PMC6812754

[3] Gao M, Piernas C, Astbury NM, Hippisley-Cox J, O’Rahilly S, Aveyard P, et al. Associations between body-mass index and COVID-19 severity in 6· 9 million people in England: a prospective, community-based, cohort study. Lancet Diabetes Endocrinol. 2021;9(6):350–9. 33932335 10.1016/S2213-8587(21)00089-9 PMC8081400

[4] Williamson EJ, Walker AJ, Bhaskaran K, Bacon S, Bates C, Morton CE, et al. Factors associated with COVID-19-related death using OpenSAFELY. Nature. 2020;584(7821):430–6. 32640463 10.1038/s41586-020-2521-4 PMC7611074

[5] Leong A, Cole JB, Brenner LN, Meigs JB, Florez JC, Mercader JM. Cardiometabolic risk factors for COVID-19 susceptibility and severity: a Mendelian randomization analysis. PLoS Med. 2021;18(3):e1003553. 33661905 10.1371/journal.pmed.1003553 PMC7971850

[6] Du Y, Lv Y, Zha W, Zhou N, Hong X. Association of body mass index (BMI) with critical COVID-19 and in-hospital mortality: a dose-response meta-analysis. Metabolism. 2021;117:154373. 32949592 10.1016/j.metabol.2020.154373 PMC7493748

[7] Gao M, Wang Q, Piernas C, Astbury NM, Jebb SA, Holmes MV, et al. Associations between body composition, fat distribution and metabolic consequences of excess adiposity with severe COVID-19 outcomes: observational study and Mendelian randomisation analysis. Int J Obes (Lond). 2022;46(5):943–50. 35031696 10.1038/s41366-021-01054-3 PMC8758930

[8] Luo S, Liang Y, Wong THT, Schooling CM, Au Yeung SL. Identifying factors contributing to increased susceptibility to COVID-19 risk: a systematic review of Mendelian randomization studies. Int J Epidemiol. 2022;51(4):1088–105. 35445260 10.1093/ije/dyac076 PMC9047195

[9] Clayton GL, Soares AG, Goulding N, Borges MC, Holmes M, Davey Smith G, et al. A framework for assessing selection and misclassification bias in mendelian randomisation studies: an illustrative example between body mass index and covid-19. BMJ. 2023;381:e072148. 10.1136/bmj-2022-072148 PMC10277657 37336561

[10] Freuer D, Linseisen J, Meisinger C. Impact of body composition on COVID-19 susceptibility and severity: a two-sample multivariable Mendelian randomization study. Metabolism. 2021;118:154732. 33631142 10.1016/j.metabol.2021.154732 PMC7900753

[11] COVID-19 Host Genetics Initiative. Mapping the human genetic architecture of COVID-19. Nature. 2021;600(7889):472–7. 34237774 10.1038/s41586-021-03767-x PMC8674144

[12] O’Brien JM Jr, Phillips GS, Ali NA, Lucarelli M, Marsh CB, Lemeshow S. Body mass index is independently associated with hospital mortality in mechanically ventilated adults with acute lung injury. Crit Care Med. 2006;34(3):738. 16521268 10.1097/01.CCM.0000202207.87891.FC PMC1868702

[13] Karampela I, Chrysanthopoulou E, Christodoulatos GS, Dalamaga M. Is there an obesity paradox in critical illness? Epidemiologic and metabolic considerations. Curr Obes Rep. 2020;9(3):231–44. 32564203 10.1007/s13679-020-00394-x

[14] Butler-Laporte G, Harroud A, Forgetta V, Richards JB. Elevated body mass index is associated with an increased risk of infectious disease admissions and mortality: a Mendelian randomization study. Clin Microbiol Infect. 2021;27(5):710–6. 10.1016/j.cmi.2020.06.014 32592749

[15] Fewell Z, Davey Smith G, Sterne JA. The impact of residual and unmeasured confounding in epidemiologic studies: a simulation study. Am J Epidemiol. 2007;166(6):646–55. 17615092 10.1093/aje/kwm165

[16] Davey Smith G, Phillips AN. Correlation without a cause: an epidemiological odyssey. Int J Epidemiol. 2020;49(1):4–14. 32244255 10.1093/ije/dyaa016

[17] Lawlor DA, Tilling K, Davey SG. Triangulation in aetiological epidemiology. Int J Epidemiol. 2016;45(6):1866–86. 28108528 10.1093/ije/dyw314 PMC5841843

[18] Munafò MR, Higgins JPT, Davey Smith G. Triangulating evidence through the inclusion of genetically informed designs. Cold Spring Harb Perspect Med. 2021;11(8):a040659. 33355252 10.1101/cshperspect.a040659 PMC8327826

[19] Brion MJA, Lawlor DA, Matijasevich A, Horta B, Anselmi L, Araújo CL, et al. What are the causal effects of breastfeeding on IQ, obesity and blood pressure? Evidence from comparing high-income with middle-income cohorts. Int J Epidemiol. 2011;40(3):670–80. 21349903 10.1093/ije/dyr020 PMC3147072

[20] Ferrando-Vivas P, Doidge J, Thomas K, Gould DW, Mouncey P, Shankar-Hari M, et al. Prognostic factors for 30-day mortality in critically ill patients with coronavirus disease 2019: an observational cohort study. Crit Care Med. 2021;49(1):102. 33116052 10.1097/CCM.0000000000004740 PMC7737692

[21] Doidge JC, Gould DW, Ferrando-Vivas P, Mouncey PR, Thomas K, Shankar-Hari M, et al. Trends in intensive care for patients with COVID-19 in England, Wales, and Northern Ireland. Am J Respir Crit Care Med. 2021;203(5):565–74. 33306946 10.1164/rccm.202008-3212OC PMC7924583

[22] Griffith GJ, Morris TT, Tudball MJ, Herbert A, Mancano G, Pike L, et al. Collider bias undermines our understanding of COVID-19 disease risk and severity. Nat Commun. 2020;11(1):1–12. 33184277 10.1038/s41467-020-19478-2 PMC7665028

[23] Richards-Belle A, Orzechowska I, Gould DW, Thomas K, Doidge JC, Mouncey PR, et al. COVID-19 in critical care: epidemiology of the first epidemic wave across England, Wales and Northern Ireland. Intensive Care Med. 2020;46(11):2035–47. 33034689 10.1007/s00134-020-06267-0 PMC7545019

[24] Harrison DA, Brady AR, Rowan K. Case mix, outcome and length of stay for admissions to adult, general critical care units in England, Wales and Northern Ireland: the Intensive Care National Audit & Research Centre Case Mix Programme Database. Crit Care. 2004;8(2):1–13. 15025784 10.1186/cc3745 PMC420043

[25] Intensive Care National Audit and Research Centre. ICNARC report on COVID-19 in critcal care: England, Wales and Northern Ireland: 6 July 2021. 2021

[26] Charani E, Gharbi M, Hickson M, Othman S, Alfituri A, Frost G, et al. Lack of weight recording in patients being administered narrow therapeutic index antibiotics: a prospective cross-sectional study. BMJ Open. 2015;5(4):e006092. 25838504 10.1136/bmjopen-2014-006092 PMC4390734

[27] Toft-Petersen AP, Wulff J, Harrison DA, Ostermann M, Margarson M, Rowan KM, et al. Exploring the impact of using measured or estimated values for height and weight on the relationship between BMI and acute hospital mortality. J Crit Care. 2018;44:196–202. 29156253 10.1016/j.jcrc.2017.11.021

[28] Harrell FE. Regression Modeling Strategies. With applications to linear models, logistic regression, and survival analysis. New York: Springer; 2001.

[29] Munafò MR, Tilling K, Taylor AE, Evans DM, Davey SG. Collider scope: when selection bias can substantially influence observed associations. Int J Epidemiol. 2018;47(1):226–35. 29040562 10.1093/ije/dyx206 PMC5837306

[30] Boyd AP, Kittelson JM, Gillen DL. Estimation of treatment effect under non-proportional hazards and conditionally independent censoring. Stat Med. 2012;31(28):3504–15. 22763957 10.1002/sim.5440 PMC3876422

[31] Xu RH, O’Quigley J. Estimating average regression effect under non-proportional hazards. Biostatistics. 2000;1(4):423–39. 12933565 10.1093/biostatistics/1.4.423

[32] Mathur R, Rentsch CT, Morton CE, Hulme WJ, Schultze A, MacKenna B, et al. Ethnic differences in SARS-CoV-2 infection and COVID-19-related hospitalisation, intensive care unit admission, and death in 17 million adults in England: an observational cohort study using the OpenSAFELY platform. Lancet. 2021;397(10286):1711–24. 33939953 10.1016/S0140-6736(21)00634-6 PMC8087292

[33] Aghili SMM, Ebrahimpur M, Arjmand B, Shadman Z, Pejman Sani M, Qorbani M, et al. Obesity in COVID-19 era, implications for mechanisms, comorbidities, and prognosis: a review and meta-analysis. Int J Obes (Lond). 2021;45(5):998–1016. 33637951 10.1038/s41366-021-00776-8 PMC7909378

[34] Abu-Raddad LJ, Chemaitelly H, Bertollini R. Severity of SARS-CoV-2 reinfections as compared with primary infections. N Engl J Med. 2021;385(26):2487–9. 34818474 10.1056/NEJMc2108120 PMC8631440

[35] Lavine JS, Bjornstad ON, Antia R. Immunological characteristics govern the transition of COVID-19 to endemicity. Science. 2021;371(6530):741–5. 33436525 10.1126/science.abe6522 PMC7932103

[36] Bernal JL, Andrews N, Gower C, Robertson C, Stowe J, Tessier E, et al. Effectiveness of the Pfizer-BioNTech and Oxford-AstraZeneca vaccines on COVID-19 related symptoms, hospital admissions, and mortality in older adults in England: test negative case-control study. BMJ. 2021;373:n1088. 33985964 10.1136/bmj.n1088 PMC8116636

[37] Millard LAC, Fernández-Sanlés A, Carter AR, Hughes RA, Tilling K, Morris TP, et al. Exploring the impact of selection bias in observational studies of COVID-19: a simulation study. Int J Epidemiol. 2023;52(1):44–57. 36474414 10.1093/ije/dyac221 PMC9908043

[38] Treskova-Schwarzbach M, Haas L, Reda S, Pilic A, Borodova A, Karimi K, et al. Pre-existing health conditions and severe COVID-19 outcomes: an umbrella review approach and meta-analysis of global evidence. BMC Med. 2021;19(1):1–26. 34446016 10.1186/s12916-021-02058-6 PMC8390115

[39] Kooistra EJ, Brinkman S, van der Voort PH, de Keizer NF, Dongelmans DA, Kox M, et al. Body mass index and mortality in coronavirus disease 2019 and other diseases: a cohort study in 35,506 ICU patients. Crit Care Med. 2022;50(1):e1. 34374504 10.1097/CCM.0000000000005216 PMC8670082

[40] Recalde M, Pistillo A, Fernandez-Bertolin S, Roel E, Aragon M, Freisling H, et al. Body Mass Index and Risk of COVID-19 Diagnosis, Hospitalization, and Death: a cohort study of 2 524 926 Catalans. J Clin Endocrinol Metab. 2021;106(12):e5030–42. 34297116 10.1210/clinem/dgab546 PMC8344917

[41] Vera-Zertuche J, Mancilla-Galindo J, Tlalpa-Prisco M, Aguilar-Alonso P, Aguirre-García M, Segura-Badilla O, et al. Obesity is a strong risk factor for short-term mortality and adverse outcomes in Mexican patients with COVID-19: a national observational study. Epidemiol Infect. 2021;149:e109. 33913410 10.1017/S0950268821001023 PMC8134888

[42] Yates T, Zaccardi F, Islam N, Razieh C, Gillies CL, Lawson CA, et al. Obesity, ethnicity, and risk of critical care, mechanical ventilation, and mortality in patients admitted to hospital with COVID-19: analysis of the ISARIC CCP-UK Cohort. Obesity (Silver Spring). 2021;29(7):1223–30. 33755331 10.1002/oby.23178 PMC8251439

[43] Espiritu AI, Reyes NGD, Leochico CFD, Sy MCC, Villanueva EQ III, Anlacan VMM, et al. Body mass index and its association with COVID-19 clinical outcomes: findings from the Philippine CORONA study. Clin Nutr ESPEN. 2022;49:402–10. 35623845 10.1016/j.clnesp.2022.03.013 PMC8968152

[44] Friedman AN, Guirguis J, Kapoor R, Gupta S, Leaf DE, Timsina LR, et al. Obesity, inflammatory and thrombotic markers, and major clinical outcomes in critically ill patients with COVID-19 in the US. Obesity (Silver Spring). 2021;29(10):1719–30. 34109768 10.1002/oby.23245

[45] Huang HK, Bukhari K, Peng CCH, Hung DP, Shih MC, Chang RHE, et al. The J-shaped relationship between body mass index and mortality in patients with COVID-19: a dose-response meta-analysis. Diabetes Obes Metab. 2021;23(7):1701. 33764660 10.1111/dom.14382 PMC8250762

[46] Paolini JBM, Mancini J, Genestal M, Gonzalez H, McKay RE, Samii K, et al. Predictive value of abdominal obesity vs. body mass index for determining risk of intensive care unit mortality. Crit Care Med. 2010;38(5):1308–14. 20228682 10.1097/CCM.0b013e3181d8cd8b

[47] Bell JA, Carslake D, O’Keeffe LM, Frysz M, Howe LD, Hamer M, et al. Associations of body mass and fat indexes with cardiometabolic traits. J Am Coll Cardiol. 2018;72(24):3142–54. 30545453 10.1016/j.jacc.2018.09.066 PMC6290112

[48] Heymsfield SB, Peterson CM, Thomas DM, Heo M, Schuna JM. Why are there race/ethnic differences in adult body mass index-adiposity relationships? A quantitative critical review. Obes Rev. 2016;17(3):262–75. 26663309 10.1111/obr.12358 PMC4968570

[49] Deurenberg P, Yap M, van Staveren WA. Body mass index and percent body fat: a metaanalysis among different ethnic groups. Int J Obes (Lond). 1998;22(12):1164–71. 10.1038/sj.ijo.0800741 9877251

[50] Stensrud MJ, Hernán MA. Why Test for Proportional Hazards? JAMA. 2020;323(14):1401–2. 32167523 10.1001/jama.2020.1267 PMC11983487

