# Supplementary material for: Adiposity and mortality among intensive care patients with COVID-19 and non-COVID-19 respiratory conditions: a cross-context comparison study in the UK
Source: BMC Med. 2024 Sep 13;22:391. doi: 10.1186/s12916-024-03598-3 (PMC11401253; doi:10.1186/s12916-024-03598-3)
Supplement: Supplementary file 4 — Additional file 4: Figure S4 Sex and ethnic group profiles of ICU patients with COVID-19 (5 Feb 2020 to 1 Aug 2021) and non-COVID-19 respiratory conditions (1 Feb 2018 to 31 Aug 2019), by geographical region. [file 12916_2024_3598_MOESM4_ESM.docx]

**Additional file 4: Figure S4** Sex and ethnic group profiles of ICU patients with COVID-19 (5 Feb 2020 to 1 Aug 2021) and non-COVID-19 respiratory conditions (1 Feb 2018 to 31 Aug 2019), by geographical region.
