# Supplementary material for: Adiposity and mortality among intensive care patients with COVID-19 and non-COVID-19 respiratory conditions: a cross-context comparison study in the UK
Source: BMC Med. 2024 Sep 13;22:391. doi: 10.1186/s12916-024-03598-3 (PMC11401253; doi:10.1186/s12916-024-03598-3)
Supplement: Supplementary file 8 — Additional file 8: Figure S8 Respiratory support, BMI reporting and mortality profiles of ICU patients with COVID-19 (1 March 2020 to 31 July 2021) and non-COVID-19 respiratory conditions (1 Feb 2018 to 31 Aug 2019), by date of admission. [file 12916_2024_3598_MOESM8_ESM.docx]

**Additional file 8: Figure S8** Respiratory support, BMI reporting and mortality profiles of ICU patients with COVID-19 (1 March 2020 to 31 July 2021) and non-COVID-19 respiratory conditions (1 Feb 2018 to 31 Aug 2019), by date of admission.

**Note:** Feb 2020 and Aug 2021 are excluded from COVID-19 profiles due to low case counts (N=5 and N=18 respectively)
