# Supplementary material for: Adiposity and mortality among intensive care patients with COVID-19 and non-COVID-19 respiratory conditions: a cross-context comparison study in the UK
Source: BMC Med. 2024 Sep 13;22:391. doi: 10.1186/s12916-024-03598-3 (PMC11401253; doi:10.1186/s12916-024-03598-3)
Supplement: Supplementary file 15 — Additional file 15: Figure S15 Association between BMI and mortality in COVID-19 and non-COVID-19 respiratory patients, by geographical region. [file 12916_2024_3598_MOESM15_ESM.docx]

**Additional file 15: Figure S15** Association between BMI and mortality in COVID-19 and non-COVID-19 respiratory patients, by geographical region.

Results from parametric survival analyses with Gompertz baseline hazard functions. BMI was modelled as a cubic spline with five knots and converted back from condition-specific standard deviations to kg/m^2^ for display. The solid line is for COVID-19 patients and the long-dashed line is for non-COVID-19 respiratory patients. Survival time was censored at 30 days with patients discharged earlier assumed to survive to 30 days. Adjusted for sex, age (cubic spline), ethnic group, deprivation and admission period.
Abbreviations: BMI; body mass index, HR; hazard ratio, CI; confidence interval. Crosses indicate the truncation of plots at the 1^st^ and 99^th^ percentiles of condition-specific BMI z-scores. Horizontal dashed lines indicate the truncation of extreme confidence limits**.**
