# Supplementary material for: Adiposity and mortality among intensive care patients with COVID-19 and non-COVID-19 respiratory conditions: a cross-context comparison study in the UK
Source: BMC Med. 2024 Sep 13;22:391. doi: 10.1186/s12916-024-03598-3 (PMC11401253; doi:10.1186/s12916-024-03598-3)
Supplement: Supplementary file 16 — Additional file 16: Table S1 Associations of confounding and selection factors with BMI and 30-day all-cause mortality among ICU patients with COVID-19 (5 Feb 2020 to 1 Aug 2021) and non-COVID-19 respiratory conditions (1 Feb 2018 to 31 Aug 2019). [file 12916_2024_3598_MOESM16_ESM.docx]

**Additional file 16: Table S1** Associations of confounding and selection factors with BMI and 30-day all-cause mortality among ICU patients with COVID-19 (5 Feb 2020 to 1 Aug 2021) and non-COVID-19 respiratory conditions (1 Feb 2018 to 31 Aug 2019).

|  | **COVID-19 patients** | **Non-COVID-19 patients** |  |
| --- | --- | --- | --- |
|  | N = 32,265 to 34,701 | N = 23,687 to 25,205 |  |
|  |  |  |  |
|  | **Mean difference (95% CI) in BMI (kg/m^2^)** | | **P_het_^a^** |
| ***Socio-demographics*** |  |  |  |
| Asian ethnicity^b^ | -2.77 (-2.99, -2.56) | -1.09 (-1.52, -0.66) | <0.0001 |
| Black ethnicity^b^ | -0.73 (-1.04, -0.41) | 0.26 (-0.29, 0.82) | 0.002 |
| White ethnicity^b^ | 2.53 (2.36, 2.71) | 0.70 (0.39, 1.01) | <0.0001 |
| Mixed/Other ethnicity^b^ | -1.59 (-1.89, -1.29) | -0.84 (-1.43, -0.26) | 0.027 |
| Deprivation (quintiles)^c^ | 0.13 (0.07, 0.19) | 0.12 (0.06, 0.19) | 0.929 |
| ***Prior or current comorbidities*** |  |  |  |
| Any past severe illness^b^ | -1.22 (-1.49, -0.95) | -0.84 (-1.07, -0.62) | 0.037 |
| Some or total dependency^b^ | 0.93 (0.68, 1.18) | 0.54 (0.34, 0.73) | 0.014 |
| Very severe cardiovascular disease^b^ | -0.34 (-1.34, 0.66) | 0.88 (0.23, 1.54) | 0.044 |
| Severe respiratory disease^b^ | 1.87 (1.08, 2.66) | 0.39 (0.00, 0.78) | 0.001 |
| Liver disease^b^ | -3.12 (-4.14, -2.10) | -2.47 (-3.11, -1.83) | 0.293 |
| End-stage renal disease^b^ | -2.03 (-2.62, -1.43) | -0.79 (-1.43, -0.16) | 0.006 |
| Metastatic disease^b^ | -2.43 (-3.44, -1.42) | -1.49 (-2.04, -0.94) | 0.107 |
| Haematological disease^b^ | -2.95 (-3.54, -2.35) | -1.24 (-1.69, -0.79) | <0.0001 |
| Immunocompromised^b^ | -2.05 (-2.46, -1.63) | -1.30 (-1.61, -0.99) | 0.005 |
| APACHE II acute severity score^c^ | -0.07 (-0.09, -0.06) | -0.08 (-0.10, -0.07) | 0.515 |
| ICNARC extreme physiology score^c^ | 0.00 (-0.02, 0.01) | -0.04 (-0.05, -0.03) | <0.0001 |
| PaO_2_/FiO_2_ ratio^c^ | -0.14 (-0.15, -0.13) | 0.00 (-0.01, 0.00) | <0.0001 |
| Advanced respiratory support (days)^c^ | 0.00 (-0.01, 0.00) | 0.01 (0.00, 0.02) | 0.063 |
|  |  |  |  |
|  | **Hazard ratio (95% CI) for mortality** | | **P_het_^a^** |
| ***Socio-demographics*** |  |  |  |
| Asian ethnicity^b^ | 1.33 (1.27, 1.39) | 1.11 (0.98, 1.25) | 0.006 |
| Black ethnicity^b^ | 1.04 (0.97, 1.12) | 0.67 (0.54, 0.82) | <0.0001 |
| White ethnicity^b^ | 0.85 (0.82, 0.89) | 1.12 (1.02, 1.23) | <0.0001 |
| Mixed/Other ethnicity^b^ | 0.87 (0.80, 0.94) | 0.78 (0.64, 0.95) | 0.300 |
| Deprivation (quintiles)^c^ | 1.04 (1.03, 1.06) | 0.99 (0.98, 1.01) | <0.0001 |
| ***Prior or current comorbidities*** |  |  |  |
| Any past severe illness^b^ | 1.48 (1.40, 1.57) | 1.71 (1.61, 1.81) | 0.001 |
| Some or total dependency^b^ | 1.36 (1.29, 1.44) | 1.40 (1.33, 1.47) | 0.503 |
| Very severe cardiovascular disease^b^ | 1.35 (1.11, 1.63) | 1.56 (1.35, 1.81) | 0.225 |
| Severe respiratory disease^b^ | 1.48 (1.27, 1.73) | 1.47 (1.33, 1.62) | 0.919 |
| Liver disease^b^ | 2.29 (1.89, 2.77) | 2.62 (2.28, 3.01) | 0.264 |
| End-stage renal disease^b^ | 1.38 (1.22, 1.57) | 0.80 (0.66, 0.98) | <0.0001 |
| Metastatic disease^b^ | 1.47 (1.22, 1.79) | 1.85 (1.64, 2.09) | 0.050 |
| Haematological disease^b^ | 1.77 (1.59, 1.97) | 1.95 (1.77, 2.15) | 0.192 |
| Immunocompromised^b^ | 1.61 (1.48, 1.74) | 1.61 (1.49, 1.73) | 0.985 |
| APACHE II acute severity score^c^ | 1.08 (1.08, 1.09) | 1.12 (1.11, 1.12) | <0.0001 |
| ICNARC extreme physiology score^c^ | 1.07 (1.06, 1.07) | 1.09 (1.09, 1.10) | <0.0001 |
| PaO_2_/FiO_2_ ratio^c^ | 0.95 (0.95, 0.96) | 0.95 (0.94, 0.95) | 0.002 |
| Advanced respiratory support (days)^c^ | 0.99 (0.99, 0.99) | 1.00 (0.99, 1.00) | <0.0001 |

Abbreviations: BMI body mass index, ICU intensive care unit, CI confidence interval
Mean differences were from linear regression and hazard ratios were from parametric survival analyses with a Gompertz-distributed baseline hazard function. Both were adjusted for sex and age (cubic splines). Analyses used all patients in the main analysis sample who had non-missing data on the covariate in question.
^a^ P-value for equality of estimates between COVID-19 and non-COVID-19 patients
^b^ Binary variables (each category of ethnicity is thus compared to all others combined)
^c^ Continuous variables
