# Supplementary material for: Adiposity and mortality among intensive care patients with COVID-19 and non-COVID-19 respiratory conditions: a cross-context comparison study in the UK
Source: BMC Med. 2024 Sep 13;22:391. doi: 10.1186/s12916-024-03598-3 (PMC11401253; doi:10.1186/s12916-024-03598-3)
Supplement: Supplementary file 17 — Additional file 17: Table S2 Associations of confounding/selection factors with BMI among ICU patients with COVID-19, by admission date [file 12916_2024_3598_MOESM17_ESM.docx]

**Additional file 17: Table S2** Associations of confounding/selection factors with BMI among ICU patients with COVID-19, by admission date

|  | **Mean difference (95% confidence interval) in BMI (kg/m^2^) among COVID-19 patients** | | | | | | **P_het_^a^** |
| --- | --- | --- | --- | --- | --- | --- | --- |
|  | **Feb-Apr 2020** | **May-Jul 2020** | **Aug-Oct 2020** | **Nov 2020-Jan 2021** | **Feb-Apr 2021** | **May-Aug 2021** |  |
|  | N = 7,801 to 8,248 | N = 1,394 to 1,506 | N = 2,747 to 2,928 | N = 14,643 to 15,828 | N = 3,851 to 4,163 | N = 1,829 to 2,028 |  |
| ***Socio-demographics*** |  |  |  |  |  |  |  |
| Asian ethnicity^b^ | -2.66 (-3.11, -2.22) | -1.49 (-2.49, -0.49) | -2.08 (-2.78, -1.37) | -2.92 (-3.23, -2.60) | -3.51 (-4.14, -2.88) | -2.80 (-3.65, -1.95) | 0.006 |
| Black ethnicity^b^ | -0.39 (-0.92, 0.13) | -0.29 (-1.99, 1.42) | -0.44 (-1.73, 0.85) | -0.80 (-1.30, -0.30) | -0.81 (-1.88, 0.26) | 0.66 (-0.45, 1.76) | 0.292 |
| White ethnicity^b^ | 2.19 (1.85, 2.53) | 1.73 (0.88, 2.58) | 2.36 (1.75, 2.97) | 2.67 (2.41, 2.93) | 2.93 (2.42, 3.45) | 1.71 (1.03, 2.38) | 0.007 |
| Mixed/Other ethnicity^b^ | -1.23 (-1.79, -0.68) | -2.12 (-3.75, -0.49) | -2.84 (-4.07, -1.61) | -1.60 (-2.05, -1.14) | -1.41 (-2.34, -0.48) | -0.59 (-1.78, 0.60) | 0.135 |
| Deprivation (quintiles)^c^ | -0.02 (-0.14, 0.09) | 0.39 (0.12, 0.67) | 0.17 (-0.02, 0.37) | 0.11 (0.03, 0.19) | 0.19 (0.02, 0.36) | 0.20 (-0.05, 0.44) | 0.052 |
| ***Prior or current comorbidities*** |  |  |  |  |  |  |  |
| Any past severe illness^b^ | -1.22 (-1.80, -0.63) | -2.08 (-3.21, -0.95) | -0.93 (-1.83, -0.03) | -1.11 (-1.52, -0.69) | -1.73 (-2.47, -0.98) | -0.89 (-1.97, 0.19) | 0.416 |
| Some or total dependency^b^ | 1.04 (0.47, 1.60) | 0.49 (-0.51, 1.50) | 0.39 (-0.41, 1.20) | 0.97 (0.60, 1.33) | 0.81 (0.13, 1.50) | 1.27 (0.04, 2.50) | 0.714 |
| Very severe cardiovascular disease^b^ | 0.61 (-1.75, 2.98) | -1.71 (-5.07, 1.65) | 1.31 (-1.40, 4.01) | -0.92 (-2.44, 0.60) | -1.73 (-4.48, 1.01) | 1.31 (-3.83, 6.45) | 0.467 |
| Severe respiratory disease^b^ | 1.27 (-0.55, 3.09) | -2.09 (-4.64, 0.46) | 1.87 (-0.39, 4.12) | 1.90 (0.64, 3.17) | 3.95 (1.81, 6.10) | 4.42 (1.36, 7.48) | 0.007 |
| Liver disease^b^ | 0.79 (-2.29, 3.88) | -5.66 (-9.00, -2.31) | -3.66 (-6.82, -0.49) | -2.97 (-4.45, -1.50) | -4.89 (-7.42, -2.36) | -4.91 (-9.10, -0.72) | 0.052 |
| End-stage renal disease^b^ | -1.59 (-2.85, -0.34) | 2.33 (-0.12, 4.78) | -1.50 (-3.63, 0.63) | -2.66 (-3.55, -1.77) | -3.03 (-4.69, -1.37) | -1.47 (-4.01, 1.08) | 0.006 |
| Metastatic disease^b^ | -0.01 (-2.46, 2.44) | -4.70 (-8.13, -1.27) | -2.25 (-5.42, 0.92) | -2.68 (-4.15, -1.21) | -4.46 (-7.22, -1.70) | 0.55 (-4.29, 5.39) | 0.106 |
| Haematological disease^b^ | -2.97 (-4.20, -1.75) | -4.67 (-6.74, -2.61) | -2.06 (-4.14, 0.03) | -2.81 (-3.74, -1.88) | -3.32 (-4.84, -1.80) | -1.54 (-4.12, 1.04) | 0.434 |
| Immunocompromised^b^ | -1.81 (-2.68, -0.93) | -3.79 (-5.57, -2.00) | -2.19 (-3.45, -0.92) | -1.73 (-2.38, -1.08) | -2.93 (-4.07, -1.79) | -1.60 (-3.22, 0.02) | 0.188 |
| APACHE II acute severity score^c^ | -0.01 (-0.04, 0.03) | -0.08 (-0.15, -0.01) | -0.02 (-0.08, 0.03) | -0.10 (-0.12, -0.07) | -0.11 (-0.15, -0.06) | -0.07 (-0.13, 0.00) | 0.0003 |
| ICNARC extreme physiology score^c^ | 0.03 (0.01, 0.05) | -0.03 (-0.08, 0.02) | 0.04 (0.00, 0.08) | -0.01 (-0.03, 0.00) | -0.01 (-0.04, 0.02) | 0.03 (-0.02, 0.08) | 0.003 |
| PaO_2_/FiO_2_ ratio^c^ | -0.10 (-0.12, -0.08) | -0.12 (-0.16, -0.09) | -0.16 (-0.19, -0.13) | -0.14 (-0.15, -0.13) | -0.15 (-0.17, -0.12) | -0.17 (-0.21, -0.14) | 0.001 |
| Advanced respiratory support (days)^c^ | -0.01 (-0.02, 0.00) | 0.02 (-0.01, 0.04) | 0.01 (0.00, 0.03) | 0.00 (-0.01, 0.01) | 0.01 (-0.01, 0.02) | 0.05 (0.01, 0.09) | 0.024 |

Abbreviations: BMI body mass index, ICU intensive care unit
Mean differences were from linear regression. Models were adjusted for sex and age (cubic splines). Analyses used all patients in the main analysis sample who had non-missing data on the covariate in question.
^a^ P-value for equality of estimates between periods. ^b^ Binary variables (each category of ethnicity is thus compared to all others combined). ^c^ Continuous variables
