# Supplementary material for: Adiposity and mortality among intensive care patients with COVID-19 and non-COVID-19 respiratory conditions: a cross-context comparison study in the UK
Source: BMC Med. 2024 Sep 13;22:391. doi: 10.1186/s12916-024-03598-3 (PMC11401253; doi:10.1186/s12916-024-03598-3)
Supplement: Supplementary file 18 — Additional file 18: Table S3 Associations of confounding/selection factors with BMI among ICU patients with COVID-19, by admission region [file 12916_2024_3598_MOESM18_ESM.docx]

**Additional file 18: Table S3** Associations of confounding/selection factors with BMI among ICU patients with COVID-19, by admission region

|  | **Mean difference (95% confidence interval) in BMI (kg/m^2^) among COVID-19 patients** | | | | | | **P_het_^a^** |
| --- | --- | --- | --- | --- | --- | --- | --- |
|  | **London, England** | **E England & Midlands** | **NE & NW England, Yorkshire** | **SE & SW England** | **Wales** | **Northern Ireland** |  |
|  | N = 7,391 to 8,014 | N = 8,113 to 8,632 | N = 9,706 to 10,657 | N = 5,138 to 5,442 | N = 1,292 to 1,328 | N = 625 to 628 |  |
| ***Socio-demographics*** |  |  |  |  |  |  |  |
| Asian ethnicity^b^ | -2.18 (-2.55, -1.81) | -2.51 (-2.93, -2.09) | -2.53 (-2.95, -2.11) | -3.69 (-4.32, -3.07) | -2.26 (-4.08, -0.44) | -8.45 (-13.32, -3.58) | 0.0004 |
| Black ethnicity^b^ | 0.92 (0.50, 1.34) | -0.42 (-1.16, 0.31) | -1.73 (-2.60, -0.86) | -1.27 (-2.39, -0.14) | -1.84 (-6.05, 2.36) | -5.19 (-11.72, 1.34) | <0.0001 |
| White ethnicity^b^ | 1.51 (1.17, 1.84) | 2.29 (1.94, 2.65) | 2.63 (2.27, 2.99) | 3.02 (2.52, 3.53) | 2.12 (0.68, 3.56) | 3.07 (0.54, 5.60) | <0.0001 |
| Mixed/Other ethnicity^b^ | -0.57 (-1.02, -0.13) | -1.38 (-2.05, -0.71) | -2.02 (-2.76, -1.28) | -1.22 (-2.14, -0.30) | -1.51 (-4.08, 1.06) | 0.00 (-3.18, 3.18) | 0.026 |
| Deprivation (quintiles)^c^ | 0.16 (0.03, 0.30) | 0.05 (-0.06, 0.16) | 0.15 (0.05, 0.26) | 0.20 (0.05, 0.34) | -0.06 (-0.34, 0.22) | 0.35 (-0.07, 0.76) | 0.286 |
| ***Prior or current comorbidities*** |  |  |  |  |  |  |  |
| Any past severe illness^b^ | -1.16 (-1.71, -0.62) | -1.02 (-1.56, -0.49) | -1.15 (-1.65, -0.66) | -1.52 (-2.23, -0.80) | -1.38 (-2.80, 0.03) | -0.78 (-2.97, 1.41) | 0.925 |
| Some or total dependency^b^ | 0.38 (-0.13, 0.90) | 1.20 (0.68, 1.72) | 0.73 (0.29, 1.17) | 1.50 (0.87, 2.13) | 0.97 (-0.17, 2.12) | 3.73 (1.53, 5.92) | 0.008 |
| Very severe cardiovascular disease^b^ | -0.11 (-2.30, 2.08) | -0.48 (-2.39, 1.42) | -0.90 (-2.47, 0.66) | 0.96 (-2.90, 4.83) | -1.74 (-6.58, 3.11) | n/a (n<5) | 0.273 |
| Severe respiratory disease^b^ | 4.26 (2.42, 6.10) | 2.43 (0.86, 4.00) | 0.41 (-0.82, 1.63) | 0.94 (-2.38, 4.26) | 1.95 (-0.93, 4.82) | 0.28 (-5.65, 6.22) | 0.024 |
| Liver disease^b^ | -3.46 (-5.30, -1.61) | -3.10 (-5.36, -0.84) | -2.18 (-3.98, -0.38) | -3.67 (-6.57, -0.77) | 0.01 (-5.12, 5.14) | n/a (n<5) | 0.309 |
| End-stage renal disease^b^ | -1.41 (-2.36, -0.45) | -1.73 (-3.09, -0.38) | -2.43 (-3.66, -1.20) | -1.63 (-3.24, -0.01) | -0.65 (-3.98, 2.69) | -5.20 (-11.15, 0.76) | 0.640 |
| Metastatic disease^b^ | -1.45 (-3.32, 0.43) | -2.69 (-4.74, -0.64) | -2.60 (-4.47, -0.73) | -2.67 (-5.23, -0.11) | -3.69 (-10.18, 2.79) | n/a (n<5) | 0.914 |
| Haematological disease^b^ | -2.52 (-3.61, -1.43) | -3.57 (-4.76, -2.37) | -2.76 (-3.92, -1.60) | -2.41 (-3.92, -0.90) | -2.49 (-5.54, 0.55) | -2.47 (-6.70, 1.75) | 0.829 |
| Immunocompromised^b^ | -1.83 (-2.65, -1.00) | -2.49 (-3.39, -1.59) | -1.84 (-2.61, -1.08) | -1.97 (-2.96, -0.97) | -3.32 (-5.38, -1.26) | 0.66 (-2.26, 3.57) | 0.282 |
| APACHE II acute severity score^c^ | -0.04 (-0.08, -0.01) | -0.05 (-0.08, -0.02) | -0.09 (-0.12, -0.06) | -0.07 (-0.11, -0.03) | -0.09 (-0.17, -0.01) | -0.08 (-0.22, 0.05) | 0.343 |
| ICNARC extreme physiology score^c^ | 0.00 (-0.02, 0.02) | 0.01 (-0.01, 0.03) | 0.02 (0.00, 0.04) | -0.01 (-0.04, 0.01) | 0.00 (-0.05, 0.05) | -0.04 (-0.12, 0.05) | 0.237 |
| PaO_2_/FiO_2_ ratio^c^ | -0.11 (-0.13, -0.09) | -0.14 (-0.16, -0.12) | -0.15 (-0.16, -0.13) | -0.16 (-0.19, -0.14) | -0.12 (-0.16, -0.08) | -0.24 (-0.31, -0.17) | <0.0001 |
| Advanced respiratory support (days)^c^ | 0.00 (-0.01, 0.01) | 0.01 (0.00, 0.02) | 0.01 (0.00, 0.02) | 0.00 (-0.01, 0.02) | -0.01 (-0.04, 0.02) | -0.01 (-0.05, 0.03) | 0.242 |

Abbreviations: BMI body mass index, ICU intensive care unit
Mean differences were from linear regression. Models were adjusted for sex and age (cubic splines). Analyses used all patients in the main analysis sample who had non-missing data on the covariate in question.
^a^ P-value for equality of estimates between regions. ^b^ Binary variables (each category of ethnicity is thus compared to all others combined). ^c^ Continuous variables
