# Supplementary material for: Adiposity and mortality among intensive care patients with COVID-19 and non-COVID-19 respiratory conditions: a cross-context comparison study in the UK
Source: BMC Med. 2024 Sep 13;22:391. doi: 10.1186/s12916-024-03598-3 (PMC11401253; doi:10.1186/s12916-024-03598-3)
Supplement: Supplementary file 19 — Additional file 19: Table S4 Associations of confounding/selection factors with BMI among ICU patients with non-COVID-19 respiratory conditions, by admission date [file 12916_2024_3598_MOESM19_ESM.docx]

**Additional file 19: Table S4** Associations of confounding/selection factors with BMI among ICU patients with non-COVID-19 respiratory conditions, by admission date

|  | **Mean difference (95% confidence interval) in BMI (kg/m^2^) among non-COVID-19 patients** | | | | | | **P_het_^a^** |
| --- | --- | --- | --- | --- | --- | --- | --- |
|  | **Feb-Apr 2018** | **May-Jul 2018** | **Aug-Oct 2018** | **Nov 2018-Jan 2019** | **Feb-Apr 2019** | **May-Aug 2019** |  |
|  | N = 4,388 to 4,654 | N = 3,099 to 3,313 | N = 2,935 to 3,169 | N = 5,037 to 5,305 | N = 4,228 to 4,480 | N = 4,000 to 4,284 |  |
| ***Socio-demographics*** |  |  |  |  |  |  |  |
| Asian ethnicity^b^ | -1.42 (-2.41, -0.43) | -1.58 (-2.85, -0.30) | -0.95 (-2.18, 0.28) | -0.73 (-1.68, 0.21) | -1.34 (-2.39, -0.30) | -0.68 (-1.65, 0.28) | 0.776 |
| Black ethnicity^b^ | 1.26 (-0.02, 2.54) | -0.34 (-1.84, 1.15) | 0.33 (-1.18, 1.84) | -1.59 (-2.86, -0.32) | 1.62 (0.22, 3.01) | 0.40 (-0.89, 1.69) | 0.011 |
| White ethnicity^b^ | 1.06 (0.35, 1.76) | 0.87 (0.01, 1.74) | 0.70 (-0.15, 1.55) | 0.59 (-0.08, 1.26) | 0.44 (-0.29, 1.18) | 0.57 (-0.15, 1.29) | 0.872 |
| Mixed/Other ethnicity^b^ | -2.75 (-4.14, -1.37) | -0.10 (-1.75, 1.55) | -1.26 (-2.86, 0.34) | 0.77 (-0.46, 2.00) | -0.78 (-2.14, 0.59) | -1.43 (-2.95, 0.09) | 0.008 |
| Deprivation (quintiles)^c^ | 0.17 (0.02, 0.32) | 0.09 (-0.09, 0.27) | 0.05 (-0.14, 0.23) | 0.03 (-0.11, 0.18) | 0.19 (0.04, 0.35) | 0.19 (0.02, 0.35) | 0.540 |
| ***Prior or current comorbidities*** |  |  |  |  |  |  |  |
| Any past severe illness^b^ | -0.44 (-0.98, 0.09) | -0.89 (-1.48, -0.29) | -1.34 (-1.96, -0.73) | -1.09 (-1.59, -0.59) | -0.49 (-1.04, 0.05) | -0.85 (-1.37, -0.32) | 0.204 |
| Some or total dependency^b^ | 0.51 (0.06, 0.97) | 0.65 (0.12, 1.18) | 0.34 (-0.21, 0.88) | 0.24 (-0.19, 0.67) | 0.74 (0.28, 1.20) | 0.79 (0.32, 1.25) | 0.496 |
| Very severe cardiovascular disease^b^ | 2.26 (0.70, 3.82) | 0.43 (-1.24, 2.11) | 0.34 (-1.55, 2.23) | 0.83 (-0.69, 2.34) | 0.76 (-0.76, 2.29) | 0.32 (-1.21, 1.86) | 0.530 |
| Severe respiratory disease^b^ | 0.77 (-0.15, 1.68) | -0.03 (-1.06, 0.99) | 0.40 (-0.66, 1.46) | -0.17 (-1.01, 0.67) | 1.03 (0.08, 1.99) | 0.46 (-0.46, 1.39) | 0.442 |
| Liver disease^b^ | -1.83 (-3.44, -0.22) | -1.51 (-3.13, 0.11) | -3.59 (-5.26, -1.93) | -2.30 (-3.82, -0.78) | -2.18 (-3.68, -0.69) | -3.12 (-4.64, -1.60) | 0.471 |
| End-stage renal disease^b^ | -1.24 (-2.66, 0.18) | 0.39 (-1.38, 2.16) | -0.50 (-2.47, 1.47) | -1.24 (-2.70, 0.22) | -1.03 (-2.51, 0.45) | -0.66 (-2.08, 0.77) | 0.750 |
| Metastatic disease^b^ | -0.86 (-2.35, 0.64) | -1.53 (-2.96, -0.10) | -1.73 (-3.09, -0.36) | -1.87 (-3.19, -0.55) | -0.86 (-2.16, 0.44) | -1.85 (-3.08, -0.62) | 0.797 |
| Haematological disease^b^ | -0.75 (-1.85, 0.35) | -1.82 (-2.96, -0.67) | -1.13 (-2.40, 0.14) | -1.12 (-2.16, -0.09) | -1.15 (-2.23, -0.07) | -1.45 (-2.46, -0.43) | 0.846 |
| Immunocompromised^b^ | -0.76 (-1.50, -0.01) | -1.28 (-2.09, -0.47) | -1.98 (-2.81, -1.15) | -1.57 (-2.26, -0.88) | -1.13 (-1.88, -0.39) | -1.17 (-1.89, -0.45) | 0.356 |
| APACHE II acute severity score^c^ | -0.09 (-0.12, -0.05) | -0.07 (-0.11, -0.03) | -0.12 (-0.16, -0.07) | -0.06 (-0.09, -0.02) | -0.06 (-0.10, -0.03) | -0.10 (-0.14, -0.07) | 0.243 |
| ICNARC extreme physiology score^c^ | -0.05 (-0.08, -0.03) | -0.04 (-0.07, -0.01) | -0.04 (-0.07, -0.01) | -0.01 (-0.03, 0.02) | -0.04 (-0.06, -0.01) | -0.06 (-0.09, -0.04) | 0.037 |
| PaO_2_/FiO_2_ ratio^c^ | -0.01 (-0.03, 0.01) | 0.01 (-0.01, 0.03) | 0.01 (-0.02, 0.03) | -0.02 (-0.04, -0.01) | -0.02 (-0.04, 0.00) | 0.02 (0.00, 0.04) | 0.014 |
| Advanced respiratory support (days)^c^ | -0.01 (-0.03, 0.01) | 0.01 (-0.02, 0.03) | 0.03 (0.00, 0.06) | 0.02 (0.00, 0.03) | 0.02 (0.00, 0.05) | -0.01 (-0.03, 0.02) | 0.179 |

Abbreviations: BMI body mass index, ICU intensive care unit
Mean differences were from linear regression. Models were adjusted for sex and age (cubic splines). Analyses used all patients in the main analysis sample who had non-missing data on the covariate in question.
^a^ P-value for equality of estimates between periods. ^b^ Binary variables (each category of ethnicity is thus compared to all others combined). ^c^ Continuous variables
