# Supplementary material for: Adiposity and mortality among intensive care patients with COVID-19 and non-COVID-19 respiratory conditions: a cross-context comparison study in the UK
Source: BMC Med. 2024 Sep 13;22:391. doi: 10.1186/s12916-024-03598-3 (PMC11401253; doi:10.1186/s12916-024-03598-3)
Supplement: Supplementary file 20 — Additional file 20: Table S5 Associations of confounding/selection factors with BMI among ICU patients with non-COVID-19 respiratory conditions, by admission region [file 12916_2024_3598_MOESM20_ESM.docx]

**Additional file 20: Table S5** Associations of confounding/selection factors with BMI among ICU patients with non-COVID-19 respiratory conditions, by admission region

|  | **Mean difference (95% confidence interval) in BMI (kg/m^2^) among non-COVID-19 patients** | | | | | | **P_het_^a^** |
| --- | --- | --- | --- | --- | --- | --- | --- |
|  | **London, England** | **E England & Midlands** | **NE & NW England, Yorkshire** | **SE & SW England** | **Wales** | **Northern Ireland** |  |
|  | N = 4,276 to 4,577 | N = 5,965 to 6,346 | N = 6,846 to 7,334 | N = 4,456 to 4,755 | N = 1,442 to 1,481 | N = 702 to 712 |  |
| ***Socio-demographics*** |  |  |  |  |  |  |  |
| Asian ethnicity^b^ | -0.68 (-1.33, -0.03) | -0.86 (-1.72, -0.01) | -1.29 (-2.31, -0.27) | -0.87 (-2.33, 0.60) | -1.72 (-5.94, 2.50) | n/a (n<5) | 0.901 |
| Black ethnicity^b^ | 0.98 (0.28, 1.68) | 0.42 (-1.04, 1.88) | 2.32 (0.34, 4.30) | -0.12 (-2.14, 1.91) | n/a (n<5) | n/a (n<5) | 0.018 |
| White ethnicity^b^ | 0.02 (-0.45, 0.48) | 0.79 (0.11, 1.46) | 0.26 (-0.55, 1.07) | 1.08 (0.06, 2.09) | 0.19 (-2.68, 3.05) | -1.49 (-6.34, 3.36) | 0.291 |
| Mixed/Other ethnicity^b^ | -0.30 (-1.08, 0.49) | -1.48 (-2.90, -0.06) | 0.74 (-0.98, 2.46) | -2.09 (-3.94, -0.24) | 1.92 (-2.29, 6.14) | 3.19 (-2.31, 8.70) | 0.077 |
| Deprivation (quintiles)^c^ | 0.27 (0.10, 0.44) | 0.14 (0.01, 0.28) | 0.01 (-0.12, 0.13) | 0.28 (0.12, 0.44) | 0.11 (-0.16, 0.38) | -0.34 (-0.74, 0.06) | 0.009 |
| ***Prior or current comorbidities*** |  |  |  |  |  |  |  |
| Any past severe illness^b^ | -0.91 (-1.41, -0.40) | -0.72 (-1.17, -0.27) | -0.98 (-1.38, -0.57) | -0.88 (-1.43, -0.33) | -0.14 (-1.04, 0.76) | -0.27 (-1.77, 1.22) | 0.602 |
| Some or total dependency^b^ | 0.08 (-0.37, 0.53) | 0.81 (0.42, 1.20) | 0.57 (0.21, 0.92) | 0.86 (0.39, 1.33) | 0.08 (-0.70, 0.86) | 0.33 (-0.88, 1.54) | 0.102 |
| Very severe cardiovascular disease^b^ | 0.73 (-0.84, 2.31) | 1.33 (0.09, 2.56) | 0.64 (-0.45, 1.72) | 1.57 (-0.68, 3.81) | 0.35 (-1.84, 2.54) | n/a (n<5) | 0.699 |
| Severe respiratory disease^b^ | -0.50 (-1.49, 0.49) | 1.58 (0.77, 2.39) | 0.01 (-0.60, 0.63) | -0.20 (-1.33, 0.92) | 1.17 (-0.17, 2.50) | 1.07 (-1.88, 4.02) | 0.008 |
| Liver disease^b^ | -2.40 (-3.99, -0.81) | -2.58 (-3.85, -1.31) | -3.05 (-4.14, -1.96) | -1.14 (-2.77, 0.48) | -1.94 (-4.43, 0.55) | -5.41 (-9.64, -1.19) | 0.335 |
| End-stage renal disease^b^ | 0.18 (-1.03, 1.40) | -0.73 (-2.00, 0.54) | -1.88 (-3.10, -0.67) | -0.80 (-2.42, 0.82) | 0.79 (-2.40, 3.97) | 0.29 (-5.26, 5.83) | 0.249 |
| Metastatic disease^b^ | -1.38 (-2.47, -0.28) | -2.01 (-3.12, -0.90) | -1.36 (-2.44, -0.28) | -0.98 (-2.27, 0.30) | -0.40 (-3.32, 2.51) | -1.31 (-4.68, 2.06) | 0.850 |
| Haematological disease^b^ | -0.79 (-1.74, 0.16) | -1.72 (-2.60, -0.83) | -1.20 (-2.03, -0.37) | -1.17 (-2.27, -0.06) | -1.05 (-3.12, 1.02) | -0.46 (-3.84, 2.91) | 0.817 |
| Immunocompromised^b^ | -1.43 (-2.12, -0.73) | -1.55 (-2.18, -0.91) | -1.44 (-2.01, -0.88) | -0.92 (-1.63, -0.22) | -0.33 (-1.56, 0.90) | -0.56 (-2.52, 1.39) | 0.422 |
| APACHE II acute severity score^c^ | -0.09 (-0.13, -0.06) | -0.07 (-0.10, -0.04) | -0.09 (-0.12, -0.06) | -0.08 (-0.12, -0.05) | -0.06 (-0.13, 0.00) | -0.10 (-0.19, -0.01) | 0.873 |
| ICNARC extreme physiology score^c^ | -0.05 (-0.08, -0.02) | -0.04 (-0.06, -0.02) | -0.03 (-0.05, -0.01) | -0.05 (-0.08, -0.03) | -0.04 (-0.09, 0.00) | -0.02 (-0.09, 0.04) | 0.845 |
| PaO_2_/FiO_2_ ratio^c^ | 0.00 (-0.02, 0.02) | 0.00 (-0.02, 0.01) | 0.00 (-0.02, 0.01) | 0.01 (-0.01, 0.03) | 0.00 (-0.04, 0.04) | -0.02 (-0.08, 0.03) | 0.792 |
| Advanced respiratory support (days)^c^ | -0.01 (-0.03, 0.01) | 0.02 (0.00, 0.04) | 0.01 (-0.01, 0.03) | 0.00 (-0.02, 0.03) | 0.01 (-0.03, 0.05) | 0.04 (-0.03, 0.11) | 0.248 |

Abbreviations: BMI body mass index, ICU intensive care unit
Mean differences were from linear regression. Models were adjusted for sex and age (cubic splines). Analyses used all patients in the main analysis sample who had non-missing data on the covariate in question.
^a^ P-value for equality of estimates between regions. ^b^ Binary variables (each category of ethnicity is thus compared to all others combined). ^c^ Continuous variables
