# Supplementary material for: Adiposity and mortality among intensive care patients with COVID-19 and non-COVID-19 respiratory conditions: a cross-context comparison study in the UK
Source: BMC Med. 2024 Sep 13;22:391. doi: 10.1186/s12916-024-03598-3 (PMC11401253; doi:10.1186/s12916-024-03598-3)
Supplement: Supplementary file 21 — Additional file 21: Table S6 Associations of confounding/selection factors with all-cause mortality among ICU patients with COVID-19, by admission date [file 12916_2024_3598_MOESM21_ESM.docx]

**Additional file 21: Table S6** Associations of confounding/selection factors with all-cause mortality among ICU patients with COVID-19, by admission date

|  | **Hazard ratio (95% confidence interval) for 30-day all-cause mortality among COVID-19 patients** | | | | | | **P_het_^a^** |
| --- | --- | --- | --- | --- | --- | --- | --- |
|  | **Feb-Apr 2020** | **May-Jul 2020** | **Aug-Oct 2020** | **Nov 2020- Jan 2021** | **Feb-Apr 2021** | **May-Aug 2021** |  |
|  | N = 7,801 to 8,248 | N = 1,394 to 1,506 | N = 2,747 to 2,928 | N = 14,643 to 15,828 | N = 3,851 to 4,163 | N = 1,829 to 2,028 |  |
| ***Socio-demographics*** |  |  |  |  |  |  |  |
| Asian ethnicity^b^ | 1.33 (1.21, 1.46) | 1.47 (1.15, 1.87) | 1.26 (1.07, 1.49) | 1.34 (1.25, 1.44) | 1.37 (1.19, 1.58) | 1.30 (0.99, 1.71) | 0.943 |
| Black ethnicity^b^ | 1.22 (1.09, 1.37) | 0.98 (0.61, 1.57) | 0.87 (0.59, 1.28) | 0.91 (0.80, 1.03) | 0.86 (0.65, 1.12) | 0.80 (0.53, 1.19) | 0.004 |
| White ethnicity^b^ | 0.78 (0.72, 0.84) | 0.76 (0.62, 0.95) | 0.94 (0.81, 1.09) | 0.87 (0.83, 0.93) | 0.95 (0.84, 1.08) | 0.99 (0.78, 1.24) | 0.020 |
| Mixed/Other ethnicity^b^ | 0.98 (0.86, 1.12) | 0.95 (0.60, 1.51) | 0.62 (0.43, 0.90) | 0.86 (0.77, 0.96) | 0.59 (0.44, 0.78) | 0.76 (0.47, 1.22) | 0.014 |
| Deprivation (quintiles)^c^ | 1.05 (1.03, 1.08) | 1.05 (0.98, 1.13) | 1.02 (0.98, 1.07) | 1.06 (1.04, 1.08) | 1.03 (0.99, 1.07) | 1.02 (0.94, 1.11) | 0.540 |
| ***Prior or current comorbidities*** |  |  |  |  |  |  |  |
| Any past severe illness^b^ | 1.24 (1.10, 1.39) | 1.73 (1.36, 2.19) | 1.68 (1.40, 2.01) | 1.55 (1.43, 1.68) | 1.59 (1.36, 1.86) | 1.90 (1.44, 2.51) | 0.004 |
| Some or total dependency^b^ | 1.38 (1.23, 1.54) | 1.30 (1.03, 1.65) | 1.56 (1.32, 1.84) | 1.35 (1.25, 1.45) | 1.35 (1.16, 1.56) | 2.11 (1.57, 2.85) | 0.058 |
| Very severe cardiovascular disease^b^ | 0.97 (0.61, 1.52) | 1.56 (0.83, 2.94) | 1.61 (0.98, 2.65) | 1.39 (1.04, 1.86) | 1.85 (1.07, 3.20) | 1.10 (0.27, 4.45) | 0.538 |
| Severe respiratory disease^b^ | 1.55 (1.13, 2.13) | 1.43 (0.82, 2.49) | 1.95 (1.30, 2.92) | 1.60 (1.26, 2.03) | 1.26 (0.79, 2.01) | 0.70 (0.29, 1.72) | 0.405 |
| Liver disease^b^ | 1.72 (0.93, 3.21) | 2.06 (1.02, 4.16) | 2.17 (1.20, 3.94) | 2.26 (1.71, 2.97) | 2.85 (1.76, 4.60) | 9.87 (5.05, 19.32) | 0.002 |
| End-stage renal disease^b^ | 1.35 (1.04, 1.74) | 0.87 (0.46, 1.63) | 1.91 (1.24, 2.95) | 1.48 (1.23, 1.77) | 1.28 (0.89, 1.84) | 1.51 (0.78, 2.93) | 0.433 |
| Metastatic disease^b^ | 1.09 (0.66, 1.81) | 1.07 (0.48, 2.41) | 2.43 (1.41, 4.20) | 1.53 (1.16, 2.02) | 1.88 (1.14, 3.11) | 2.01 (0.74, 5.44) | 0.300 |
| Haematological disease^b^ | 1.28 (1.02, 1.61) | 2.61 (1.81, 3.77) | 1.42 (0.95, 2.13) | 2.03 (1.72, 2.39) | 2.02 (1.53, 2.67) | 2.17 (1.29, 3.64) | 0.005 |
| Immunocompromised^b^ | 1.30 (1.10, 1.55) | 2.20 (1.56, 3.10) | 1.79 (1.41, 2.28) | 1.62 (1.43, 1.83) | 1.70 (1.35, 2.14) | 2.67 (1.88, 3.78) | 0.003 |
| APACHE II acute severity score^c^ | 1.07 (1.06, 1.08) | 1.10 (1.09, 1.12) | 1.09 (1.07, 1.10) | 1.08 (1.08, 1.09) | 1.09 (1.08, 1.10) | 1.13 (1.10, 1.15) | <0.0001 |
| ICNARC extreme physiology score^c^ | 1.06 (1.05, 1.06) | 1.08 (1.07, 1.09) | 1.08 (1.07, 1.09) | 1.06 (1.06, 1.07) | 1.07 (1.06, 1.08) | 1.09 (1.07, 1.11) | <0.0001 |
| PaO_2_/FiO_2_ ratio^c^ | 0.96 (0.95, 0.96) | 0.94 (0.93, 0.96) | 0.96 (0.95, 0.97) | 0.95 (0.94, 0.95) | 0.95 (0.94, 0.96) | 0.93 (0.92, 0.95) | 0.023 |
| Advanced respiratory support (days)^c^ | 0.97 (0.97, 0.97) | 0.99 (0.98, 0.99) | 0.99 (0.99, 1.00) | 0.99 (0.99, 0.99) | 0.99 (0.99, 1.00) | 1.02 (1.01, 1.03) | <0.0001 |

Abbreviations: ICU intensive care unit
Hazard ratios were from parametric survival analyses with a Gompertz-distributed baseline hazard function. Models were adjusted for sex and age (cubic splines). Analyses used all patients in the main analysis sample who had non-missing data on the covariate in question.
^a^ P-value for equality of estimates between periods. ^b^ Binary variables (each category of ethnicity is thus compared to all others combined). ^c^ Continuous variables
