# Supplementary material for: Adiposity and mortality among intensive care patients with COVID-19 and non-COVID-19 respiratory conditions: a cross-context comparison study in the UK
Source: BMC Med. 2024 Sep 13;22:391. doi: 10.1186/s12916-024-03598-3 (PMC11401253; doi:10.1186/s12916-024-03598-3)
Supplement: Supplementary file 22 — Additional file 22: Table S7 Associations of confounding/selection factors with all-cause mortality among ICU patients with COVID-19, by admission region [file 12916_2024_3598_MOESM22_ESM.docx]

**Additional file 22: Table S7** Associations of confounding/selection factors with all-cause mortality among ICU patients with COVID-19, by admission region

|  | **Hazard ratio (95% confidence interval) for 30-day all-cause mortality among COVID-19 patients** | | | | | | **P_het_^a^** |
| --- | --- | --- | --- | --- | --- | --- | --- |
|  | **London, England** | **E England & Midlands** | **NE & NW England, Yorkshire** | **SE & SW England** | **Wales** | **Northern Ireland** |  |
|  | N = 7,391 to 8,014 | N = 8,113 to 8,632 | N = 9,706 to 10,657 | N = 5,138 to 5,442 | N = 1,292 to 1,328 | N = 625 to 628 |  |
| ***Socio-demographics*** |  |  |  |  |  |  |  |
| Asian ethnicity^b^ | 1.42 (1.31, 1.55) | 1.27 (1.16, 1.39) | 1.31 (1.19, 1.44) | 1.38 (1.19, 1.60) | 1.06 (0.70, 1.61) | 1.14 (0.28, 4.67) | 0.403 |
| Black ethnicity^b^ | 1.04 (0.94, 1.15) | 1.14 (0.96, 1.35) | 0.95 (0.76, 1.20) | 1.11 (0.82, 1.49) | 1.04 (0.43, 2.51) | 1.03 (0.14, 7.41) | 0.887 |
| White ethnicity^b^ | 0.80 (0.74, 0.87) | 0.85 (0.78, 0.92) | 0.85 (0.78, 0.92) | 0.78 (0.69, 0.88) | 0.92 (0.66, 1.27) | 0.84 (0.42, 1.68) | 0.790 |
| Mixed/Other ethnicity^b^ | 0.83 (0.74, 0.92) | 0.85 (0.72, 1.01) | 0.87 (0.71, 1.06) | 1.06 (0.83, 1.34) | 1.16 (0.65, 2.06) | 1.22 (0.53, 2.80) | 0.417 |
| Deprivation (quintiles)^c^ | 1.04 (1.00, 1.07) | 1.04 (1.01, 1.06) | 1.04 (1.02, 1.07) | 1.04 (1.00, 1.08) | 1.06 (1.00, 1.13) | 0.99 (0.89, 1.09) | 0.890 |
| ***Prior or current comorbidities*** |  |  |  |  |  |  |  |
| Any past severe illness^b^ | 1.29 (1.15, 1.45) | 1.59 (1.43, 1.76) | 1.51 (1.37, 1.66) | 1.57 (1.35, 1.82) | 1.35 (1.02, 1.77) | 2.02 (1.34, 3.05) | 0.065 |
| Some or total dependency^b^ | 1.38 (1.24, 1.53) | 1.20 (1.08, 1.34) | 1.43 (1.31, 1.57) | 1.48 (1.29, 1.69) | 1.38 (1.10, 1.72) | 1.33 (0.80, 2.21) | 0.183 |
| Very severe cardiovascular disease^b^ | 1.18 (0.77, 1.82) | 1.37 (0.95, 1.98) | 1.26 (0.93, 1.71) | 2.67 (1.38, 5.14) | 0.92 (0.34, 2.48) | n/a (n<5) | 0.371 |
| Severe respiratory disease^b^ | 1.86 (1.33, 2.58) | 1.34 (0.99, 1.82) | 1.30 (1.01, 1.66) | 2.87 (1.54, 5.35) | 1.22 (0.70, 2.13) | 3.11 (1.15, 8.42) | 0.071 |
| Liver disease^b^ | 2.37 (1.66, 3.38) | 2.37 (1.56, 3.61) | 1.88 (1.33, 2.64) | 3.04 (1.72, 5.39) | 3.46 (1.53, 7.81) | n/a (n<5) | 0.645 |
| End-stage renal disease^b^ | 1.25 (1.02, 1.53) | 1.59 (1.21, 2.10) | 1.38 (1.07, 1.78) | 1.34 (0.93, 1.92) | 1.95 (1.07, 3.55) | 2.02 (0.74, 5.51) | 0.580 |
| Metastatic disease^b^ | 0.98 (0.64, 1.49) | 1.52 (1.04, 2.22) | 1.76 (1.26, 2.47) | 2.40 (1.54, 3.73) | 0.78 (0.19, 3.14) | n/a (n<5) | 0.082 |
| Haematological disease^b^ | 1.60 (1.29, 1.98) | 1.84 (1.49, 2.28) | 1.79 (1.46, 2.20) | 1.92 (1.45, 2.53) | 2.12 (1.30, 3.45) | 1.43 (0.63, 3.23) | 0.835 |
| Immunocompromised^b^ | 1.30 (1.09, 1.55) | 1.77 (1.51, 2.09) | 1.81 (1.56, 2.08) | 1.66 (1.36, 2.03) | 1.38 (0.93, 2.05) | 1.72 (0.99, 2.99) | 0.071 |
| APACHE II acute severity score^c^ | 1.08 (1.07, 1.08) | 1.09 (1.08, 1.10) | 1.08 (1.07, 1.09) | 1.09 (1.08, 1.10) | 1.07 (1.05, 1.08) | 1.11 (1.08, 1.15) | 0.004 |
| ICNARC extreme physiology score^c^ | 1.07 (1.06, 1.07) | 1.07 (1.06, 1.07) | 1.07 (1.07, 1.08) | 1.07 (1.07, 1.08) | 1.06 (1.05, 1.07) | 1.06 (1.04, 1.08) | 0.054 |
| PaO_2_/FiO_2_ ratio^c^ | 0.96 (0.95, 0.96) | 0.95 (0.95, 0.96) | 0.95 (0.94, 0.95) | 0.95 (0.95, 0.96) | 0.96 (0.95, 0.97) | 0.93 (0.91, 0.96) | 0.099 |
| Advanced respiratory support (days)^c^ | 0.98 (0.98, 0.98) | 0.99 (0.99, 0.99) | 0.99 (0.99, 1.00) | 0.99 (0.99, 0.99) | 0.98 (0.98, 0.99) | 0.98 (0.97, 0.99) | <0.0001 |

Abbreviations: ICU intensive care unit
Hazard ratios were from parametric survival analyses with a Gompertz-distributed baseline hazard function. Models were adjusted for sex and age (cubic splines). Analyses used all patients in the main analysis sample who had non-missing data on the covariate in question.
^a^ P-value for equality of estimates between regions. ^b^ Binary variables (each category of ethnicity is thus compared to all others combined). ^c^ Continuous variables
