# Supplementary material for: Adiposity and mortality among intensive care patients with COVID-19 and non-COVID-19 respiratory conditions: a cross-context comparison study in the UK
Source: BMC Med. 2024 Sep 13;22:391. doi: 10.1186/s12916-024-03598-3 (PMC11401253; doi:10.1186/s12916-024-03598-3)
Supplement: Supplementary file 23 — Additional file 23: Table S8 Associations of confounding/selection factors with all-cause mortality among ICU patients with non-COVID-19 respiratory conditions, by admission date [file 12916_2024_3598_MOESM23_ESM.docx]

**Additional file 23: Table S8** Associations of confounding/selection factors with all-cause mortality among ICU patients with non-COVID-19 respiratory conditions, by admission date

|  | **Hazard ratio (95% confidence interval) for 30-day all-cause mortality among non-COVID-19 patients** | | | | | | **P_het_^a^** |
| --- | --- | --- | --- | --- | --- | --- | --- |
|  | **Feb-Apr 2018** | **May-Jul 2018** | **Aug-Oct 2018** | **Nov 2018- Jan 2019** | **Feb-Apr 2019** | **May-Aug 2019** |  |
|  | N = 4,388 to 4,654 | N = 3,099 to 3,313 | N = 2,935 to 3,169 | N = 5,037 to 5,305 | N = 4,228 to 4,480 | N = 4,000 to 4,284 |  |
| ***Socio-demographics*** |  |  |  |  |  |  |  |
| Asian ethnicity^b^ | 1.09 (0.82, 1.45) | 1.36 (0.98, 1.89) | 1.28 (0.91, 1.79) | 0.99 (0.75, 1.32) | 0.97 (0.71, 1.33) | 1.07 (0.81, 1.42) | 0.631 |
| Black ethnicity^b^ | 0.73 (0.47, 1.12) | 0.42 (0.21, 0.84) | 0.62 (0.34, 1.13) | 0.71 (0.45, 1.14) | 0.70 (0.42, 1.17) | 0.72 (0.46, 1.14) | 0.823 |
| White ethnicity^b^ | 1.06 (0.86, 1.32) | 1.08 (0.83, 1.42) | 0.94 (0.73, 1.21) | 1.24 (0.99, 1.54) | 1.26 (0.99, 1.60) | 1.14 (0.91, 1.42) | 0.563 |
| Mixed/Other ethnicity^b^ | 0.92 (0.59, 1.44) | 0.82 (0.48, 1.39) | 1.18 (0.76, 1.84) | 0.61 (0.38, 0.97) | 0.63 (0.38, 1.03) | 0.65 (0.37, 1.15) | 0.296 |
| Deprivation (quintiles)^c^ | 0.98 (0.94, 1.02) | 1.05 (1.00, 1.11) | 0.97 (0.92, 1.02) | 1.02 (0.98, 1.07) | 0.97 (0.93, 1.01) | 0.97 (0.93, 1.02) | 0.101 |
| ***Prior or current comorbidities*** |  |  |  |  |  |  |  |
| Any past severe illness^b^ | 1.52 (1.33, 1.74) | 1.62 (1.38, 1.89) | 1.94 (1.65, 2.27) | 1.66 (1.46, 1.88) | 1.83 (1.60, 2.10) | 1.78 (1.56, 2.04) | 0.197 |
| Some or total dependency^b^ | 1.41 (1.25, 1.59) | 1.41 (1.22, 1.63) | 1.38 (1.19, 1.61) | 1.41 (1.25, 1.58) | 1.50 (1.32, 1.70) | 1.27 (1.12, 1.45) | 0.658 |
| Very severe cardiovascular disease^b^ | 0.91 (0.60, 1.39) | 1.34 (0.91, 1.99) | 1.21 (0.76, 1.93) | 2.17 (1.59, 2.97) | 2.70 (2.00, 3.63) | 1.29 (0.89, 1.88) | 0.0001 |
| Severe respiratory disease^b^ | 1.35 (1.07, 1.70) | 1.48 (1.14, 1.92) | 1.59 (1.22, 2.09) | 1.35 (1.09, 1.68) | 1.59 (1.26, 2.00) | 1.55 (1.23, 1.96) | 0.837 |
| Liver disease^b^ | 2.74 (1.95, 3.86) | 2.44 (1.68, 3.54) | 2.76 (1.94, 3.94) | 2.03 (1.41, 2.90) | 2.32 (1.67, 3.22) | 3.65 (2.68, 4.98) | 0.206 |
| End-stage renal disease^b^ | 0.79 (0.51, 1.23) | 0.70 (0.39, 1.27) | 0.84 (0.45, 1.57) | 0.91 (0.59, 1.42) | 0.71 (0.44, 1.17) | 0.84 (0.54, 1.30) | 0.976 |
| Metastatic disease^b^ | 2.04 (1.49, 2.79) | 1.54 (1.10, 2.15) | 2.25 (1.69, 3.00) | 2.09 (1.59, 2.75) | 1.76 (1.32, 2.36) | 1.59 (1.20, 2.11) | 0.386 |
| Haematological disease^b^ | 1.59 (1.24, 2.06) | 2.03 (1.58, 2.60) | 1.89 (1.42, 2.52) | 1.66 (1.31, 2.11) | 2.21 (1.75, 2.79) | 2.33 (1.88, 2.88) | 0.160 |
| Immunocompromised^b^ | 1.49 (1.25, 1.79) | 1.63 (1.34, 1.98) | 1.78 (1.45, 2.18) | 1.66 (1.41, 1.96) | 1.45 (1.20, 1.74) | 1.65 (1.39, 1.97) | 0.688 |
| APACHE II acute severity score^c^ | 1.11 (1.10, 1.12) | 1.11 (1.10, 1.13) | 1.12 (1.11, 1.13) | 1.12 (1.11, 1.13) | 1.12 (1.11, 1.13) | 1.12 (1.11, 1.13) | 0.773 |
| ICNARC extreme physiology score^c^ | 1.09 (1.08, 1.10) | 1.09 (1.08, 1.10) | 1.10 (1.09, 1.11) | 1.10 (1.10, 1.11) | 1.09 (1.08, 1.10) | 1.09 (1.09, 1.10) | 0.021 |
| PaO_2_/FiO_2_ ratio^c^ | 0.94 (0.94, 0.95) | 0.95 (0.94, 0.95) | 0.94 (0.93, 0.95) | 0.94 (0.94, 0.95) | 0.95 (0.95, 0.96) | 0.95 (0.94, 0.96) | 0.241 |
| Advanced respiratory support (days)^c^ | 0.99 (0.99, 1.00) | 1.00 (0.99, 1.00) | 1.00 (0.99, 1.01) | 1.00 (0.99, 1.00) | 1.00 (0.99, 1.01) | 1.00 (0.99, 1.01) | 0.702 |

Abbreviations: ICU intensive care unit
Hazard ratios were from parametric survival analyses with a Gompertz-distributed baseline hazard function. Models were adjusted for sex and age (cubic splines). Analyses used all patients in the main analysis sample who had non-missing data on the covariate in question.
^a^ P-value for equality of estimates between periods. ^b^ Binary variables (each category of ethnicity is thus compared to all others combined). ^c^ Continuous variables
