# Supplementary material for: Adiposity and mortality among intensive care patients with COVID-19 and non-COVID-19 respiratory conditions: a cross-context comparison study in the UK
Source: BMC Med. 2024 Sep 13;22:391. doi: 10.1186/s12916-024-03598-3 (PMC11401253; doi:10.1186/s12916-024-03598-3)
Supplement: Supplementary file 24 — Additional file 24: Table S9 Associations of confounding/selection factors with all-cause mortality among ICU patients with non-COVID-19 respiratory conditions, by admission region [file 12916_2024_3598_MOESM24_ESM.docx]

**Additional file 24: Table S9** Associations of confounding/selection factors with all-cause mortality among ICU patients with non-COVID-19 respiratory conditions, by admission region

|  | **Hazard ratio (95% confidence interval) for 30-day all-cause mortality among non-COVID-19 patients** | | | | | | **P_het_^a^** |
| --- | --- | --- | --- | --- | --- | --- | --- |
|  | **London, England** | **E England & Midlands** | **NE & NW England, Yorkshire** | **SE & SW England** | **Wales** | **Northern Ireland** |  |
|  | N = 4,276 to 4,577 | N = 5,965 to 6,346 | N = 6,846 to 7,334 | N = 4,456 to 4,755 | N = 1,442 to 1,481 | N = 702 to 712 |  |
| ***Socio-demographics*** |  |  |  |  |  |  |  |
| Asian ethnicity^b^ | 1.14 (0.94, 1.39) | 1.22 (0.96, 1.55) | 1.16 (0.87, 1.55) | 1.30 (0.85, 1.99) | 1.26 (0.40, 3.96) | n/a (n<5) | 0.980 |
| Black ethnicity^b^ | 0.76 (0.59, 0.99) | 0.67 (0.39, 1.16) | 0.77 (0.40, 1.49) | 0.60 (0.25, 1.44) | n/a (n<5) | n/a (n<5) | 0.112 |
| White ethnicity^b^ | 1.06 (0.91, 1.22) | 0.96 (0.79, 1.18) | 0.97 (0.77, 1.24) | 1.08 (0.76, 1.52) | 1.39 (0.52, 3.74) | 0.97 (0.24, 3.95) | 0.949 |
| Mixed/Other ethnicity^b^ | 0.92 (0.71, 1.19) | 0.85 (0.52, 1.39) | 0.87 (0.50, 1.50) | 0.62 (0.29, 1.30) | n/a (none died) | 0.68 (0.09, 4.89) | 0.958 |
| Deprivation (quintiles)^c^ | 0.94 (0.90, 0.99) | 1.00 (0.97, 1.04) | 0.99 (0.95, 1.02) | 0.99 (0.94, 1.04) | 0.93 (0.87, 1.00) | 1.00 (0.88, 1.14) | 0.309 |
| ***Prior or current comorbidities*** |  |  |  |  |  |  |  |
| Any past severe illness^b^ | 1.89 (1.65, 2.17) | 1.64 (1.47, 1.84) | 1.59 (1.44, 1.76) | 1.84 (1.60, 2.12) | 1.82 (1.48, 2.24) | 1.46 (0.97, 2.19) | 0.266 |
| Some or total dependency^b^ | 1.56 (1.37, 1.78) | 1.32 (1.19, 1.47) | 1.40 (1.27, 1.53) | 1.44 (1.27, 1.65) | 1.28 (1.05, 1.56) | 1.13 (0.79, 1.63) | 0.306 |
| Very severe cardiovascular disease^b^ | 1.67 (1.16, 2.40) | 1.35 (1.01, 1.80) | 1.63 (1.28, 2.06) | 1.55 (0.93, 2.59) | 1.36 (0.82, 2.24) | n/a (n<5) | 0.862 |
| Severe respiratory disease^b^ | 1.71 (1.32, 2.22) | 1.28 (1.04, 1.58) | 1.36 (1.16, 1.59) | 1.91 (1.46, 2.49) | 1.21 (0.87, 1.69) | 1.38 (0.64, 2.96) | 0.120 |
| Liver disease^b^ | 2.58 (1.81, 3.69) | 2.26 (1.69, 3.03) | 2.92 (2.33, 3.65) | 2.52 (1.74, 3.66) | 1.78 (0.99, 3.18) | 4.32 (1.88, 9.93) | 0.416 |
| End-stage renal disease^b^ | 0.93 (0.64, 1.35) | 0.89 (0.61, 1.30) | 0.88 (0.61, 1.26) | 0.29 (0.12, 0.70) | 0.53 (0.17, 1.67) | 5.18 (1.84, 14.58) | 0.003 |
| Metastatic disease^b^ | 1.74 (1.34, 2.26) | 1.61 (1.25, 2.07) | 2.11 (1.69, 2.62) | 2.00 (1.50, 2.65) | 2.89 (1.72, 4.85) | 1.79 (0.79, 4.07) | 0.345 |
| Haematological disease^b^ | 2.05 (1.65, 2.56) | 2.04 (1.68, 2.47) | 1.77 (1.47, 2.13) | 2.06 (1.61, 2.64) | 2.43 (1.65, 3.59) | 1.39 (0.61, 3.18) | 0.620 |
| Immunocompromised^b^ | 1.66 (1.38, 1.99) | 1.64 (1.41, 1.91) | 1.37 (1.19, 1.58) | 1.97 (1.66, 2.33) | 1.89 (1.45, 2.45) | 0.95 (0.52, 1.74) | 0.010 |
| APACHE II acute severity score^c^ | 1.12 (1.11, 1.13) | 1.11 (1.10, 1.12) | 1.12 (1.11, 1.13) | 1.12 (1.11, 1.13) | 1.10 (1.08, 1.11) | 1.16 (1.13, 1.20) | 0.005 |
| ICNARC extreme physiology score^c^ | 1.10 (1.09, 1.11) | 1.09 (1.09, 1.10) | 1.10 (1.09, 1.10) | 1.10 (1.09, 1.10) | 1.08 (1.07, 1.09) | 1.12 (1.10, 1.14) | 0.036 |
| PaO_2_/FiO_2_ ratio^c^ | 0.95 (0.95, 0.96) | 0.95 (0.95, 0.96) | 0.94 (0.94, 0.95) | 0.94 (0.93, 0.95) | 0.94 (0.93, 0.95) | 0.93 (0.91, 0.95) | 0.022 |
| Advanced respiratory support (days)^c^ | 1.00 (0.99, 1.01) | 1.00 (0.99, 1.00) | 0.99 (0.98, 1.00) | 1.01 (1.00, 1.01) | 0.99 (0.98, 1.00) | 1.02 (1.00, 1.04) | 0.001 |

Abbreviations: ICU intensive care unit
Hazard ratios were from parametric survival analyses with a Gompertz-distributed baseline hazard function. Models were adjusted for sex and age (cubic splines). Analyses used all patients in the main analysis sample who had non-missing data on the covariate in question.
^a^ P-value for equality of estimates between regions. ^b^ Binary variables (each category of ethnicity is thus compared to all others combined). ^c^ Continuous variables.
