# Supplementary material for: Adiposity and mortality among intensive care patients with COVID-19 and non-COVID-19 respiratory conditions: a cross-context comparison study in the UK
Source: BMC Med. 2024 Sep 13;22:391. doi: 10.1186/s12916-024-03598-3 (PMC11401253; doi:10.1186/s12916-024-03598-3)
Supplement: Supplementary file 26 — Additional file 26: Supplementary Table S11 Main analyses of all-cause mortality and BMI, restricted to ICU patients who were not physically dependent on others for the activities of daily living prior to admission. [file 12916_2024_3598_MOESM26_ESM.docx]

**Additional file 26: Supplementary Table S11** Main analyses of all-cause mortality and BMI, restricted to ICU patients who were not physically dependent on others for the activities of daily living prior to admission.

|  | **Hazard ratio (95% CI) for 30-day all-cause mortality** | |
| --- | --- | --- |
|  | **COVID-19 patients^a^** | **Non-COVID-19 patients, before pandemic^b^** |
| Deaths (N) | 10,080 (30,526) | 3,265 (16,630) |
|  |  |  |
| Per SD higher BMI | 1.04 (1.01, 1.06) | 0.82 (0.79, 0.86) |
| Underweight (<18.5 kg/m^2^) | 1.37 (1.08, 1.75) | 1.42 (1.22, 1.66) |
| Recommended (18.5-<25 kg/m^2^) | 1.00 (reference) | 1.00 (reference) |
| Overweight (25-<30 kg/m^2^) | 1.01 (0.96, 1.06) | 0.83 (0.76, 0.90) |
| Obesity 1 (30-<35 kg/m^2^) | 0.96 (0.90, 1.01) | 0.71 (0.64, 0.79) |
| Obesity 2 (35-<40 kg/m^2^) | 0.97 (0.90, 1.04) | 0.66 (0.56, 0.78) |
| Obesity 3+ (≥40 kg/m^2^) | 1.19 (1.09, 1.29) | 0.72 (0.60, 0.87) |

Abbreviations: BMI body mass index, ICU intensive care unit, CI confidence interval, SD standard deviation
Results from parametric survival analyses with Gompertz baseline hazard functions. Survival time was censored at 30 days with patients discharged earlier assumed to survive to 30 days. Adjusted for sex, age (cubic spline), ethnic group, deprivation, admission period and admission region.
^a^ COVID-19 patients admitted between 5^th^ February 2020 and 1^st^ August 2021
^b^ Non-COVID-19 respiratory patients admitted between 1^st^ February 2018 and 31^st^ August 2019
