# Supplementary material for: Adiposity and mortality among intensive care patients with COVID-19 and non-COVID-19 respiratory conditions: a cross-context comparison study in the UK
Source: BMC Med. 2024 Sep 13;22:391. doi: 10.1186/s12916-024-03598-3 (PMC11401253; doi:10.1186/s12916-024-03598-3)
Supplement: Supplementary file 27 — Additional file 27: Table S12 Number of deaths and total sample size for each BMI category in Table 2. [file 12916_2024_3598_MOESM27_ESM.docx]

**Additional file 27: Table S12** Number of deaths and total sample size for each BMI category in Table 2.

|  | **Number of deaths within 30 days of admission to ICU (total sample size)** | | | |
| --- | --- | --- | --- | --- |
|  | **COVID-19 patients^a^** | **Non-COVID-19 patients, before pandemic^b^** | **Non-COVID-19 patients, during pandemic^c^** | **Non-COVID-19 patients, excluding bacterial pneumonia^d^** |
|  |  |  |  |  |
| All BMI categories | 11,912 (34,701) | 5,637 (25,205) | 1,892 (8,241) | 4,452 (19,535) |
| Underweight (<18.5 kg/m^2^) | 100 (280) | 405 (1,334) | 113 (398) | 318 (1,020) |
| Recommended (18.5-<25 kg/m^2^) | 2,708 (7,181) | 2,309 (9,323) | 716 (2,855) | 1,817 (7,201) |
| Overweight (25-<30 kg/m^2^) | 4,115 (11,069) | 1,666 (7,475) | 568 (2,430) | 1,298 (5,707) |
| Obesity 1 (30-<35 kg/m^2^) | 2,653 (8,142) | 709 (3,763) | 260 (1,303) | 577 (2,968) |
| Obesity 2 (35-<40 kg/m^2^) | 1,248 (4,237) | 303 (1,743) | 118 (645) | 250 (1,383) |
| Obesity 3+ (≥40 kg/m^2^) | 1,088 (3,792) | 245 (1,567) | 117 (610) | 192 (1,256) |

Abbreviations: BMI body mass index, ICU intensive care unit

^a^ COVID-19 patients admitted between 5^th^ February 2020 and 1^st^ August 2021
^b^ Non-COVID-19 respiratory patients admitted between 1^st^ February 2018 and 31^st^ August 2019
^c^ Non-COVID-19 respiratory patients admitted between 1^st^ February 2020 and 30^th^ June 2021
^d^ Non-COVID-19 respiratory patients, excluding bacterial pneumonia, admitted between 1^st^ February 2018 and 31^st^ August 2019
