# Supplementary material for: Adiposity and mortality among intensive care patients with COVID-19 and non-COVID-19 respiratory conditions: a cross-context comparison study in the UK
Source: BMC Med. 2024 Sep 13;22:391. doi: 10.1186/s12916-024-03598-3 (PMC11401253; doi:10.1186/s12916-024-03598-3)
Supplement: Supplementary file 28 — Additional file 28: Table S13 Number of deaths and total sample size for each BMI category and admission period in Table 3. [file 12916_2024_3598_MOESM28_ESM.docx]

**Additional file 28: Table S13** Number of deaths and total sample size for each BMI category and admission period in Table 3.

|  | **Number of deaths within 30 days of admission to ICU (total sample size)** | | | | | |
| --- | --- | --- | --- | --- | --- | --- |
|  | **Feb-Apr** | **May-Jul** | **Aug-Oct** | **Nov-Jan** | **Feb-Apr** | **May-Aug** |
|  |  |  |  |  |  |  |
| **COVID-19 patients admitted 5^th^ February 2020 to 1^st^ August 2021** | | | | | | |
| All BMI categories | 3,173 (8,248) | 431 (1,506) | 993 (2,928) | 5,673 (15,828) | 1,313 (4,163) | 329 (2,028) |
| Underweight (<18.5 kg/m^2^) | 19 (43) | 10 (34) | 8 (23) | 34 (96) | 18 (52) | 11 (32) |
| Recommended (18.5-<25 kg/m^2^) | 807 (2,046) | 136 (408) | 217 (573) | 1,201 (3,047) | 282 (766) | 65 (341) |
| Overweight (25-<30 kg/m^2^) | 1,194 (2,905) | 139 (457) | 376 (991) | 1,906 (4,923) | 399 (1,178) | 101 (615) |
| Obesity 1 (30-<35 kg/m^2^) | 641 (1,795) | 79 (295) | 201 (662) | 1,355 (3,916) | 306 (980) | 71 (494) |
| Obesity 2 (35-<40 kg/m^2^) | 285 (823) | 37 (169) | 110 (368) | 615 (1,973) | 154 (623) | 47 (281) |
| Obesity 3+ (≥40 kg/m^2^) | 227 (636) | 30 (143) | 81 (311) | 562 (1,873) | 154 (564) | 34 (265) |
|  |  |  |  |  |  |  |
| **Non-COVID-19 patients admitted 1^st^ February 2018 to 31^st^ August 2019** | | | | | | |
| All BMI categories | 1,071 (4,654) | 750 (3,313) | 683 (3,169) | 1,182 (5,305) | 997 (4,480) | 954 (4,284) |
| Underweight (<18.5 kg/m^2^) | 79 (249) | 54 (176) | 47 (160) | 83 (291) | 59 (223) | 83 (235) |
| Recommended (18.5-<25 kg/m^2^) | 462 (1,802) | 298 (1,214) | 294 (1,167) | 446 (1,921) | 410 (1,642) | 399 (1,577) |
| Overweight (25-<30 kg/m^2^) | 308 (1,363) | 240 (1,044) | 190 (954) | 350 (1,523) | 312 (1,356) | 266 (1,235) |
| Obesity 1 (30-<35 kg/m^2^) | 140 (695) | 77 (462) | 85 (482) | 164 (837) | 121 (655) | 122 (632) |
| Obesity 2 (35-<40 kg/m^2^) | 47 (298) | 39 (217) | 41 (206) | 76 (390) | 57 (324) | 43 (308) |
| Obesity 3+ (≥40 kg/m^2^) | 35 (247) | 42 (200) | 26 (200) | 63 (343) | 38 (280) | 41 (297) |

Abbreviations: BMI body mass index, ICU intensive care unit
