# Supplementary material for: Adiposity and mortality among intensive care patients with COVID-19 and non-COVID-19 respiratory conditions: a cross-context comparison study in the UK
Source: BMC Med. 2024 Sep 13;22:391. doi: 10.1186/s12916-024-03598-3 (PMC11401253; doi:10.1186/s12916-024-03598-3)
Supplement: Supplementary file 29 — Additional file 29: Table S14 Number of deaths and total sample size for each BMI category and admission region in Table 4. [file 12916_2024_3598_MOESM29_ESM.docx]

**Additional file 29: Table S14** Number of deaths and total sample size for each BMI category and admission region in Table 4.

|  | **Number of deaths within 30 days of admission to ICU (total sample size)** | | | | | |
| --- | --- | --- | --- | --- | --- | --- |
|  | **London, England** | **E England & Midlands** | **NE & NW England, Yorkshire** | **SE & SW England** | **Wales** | **Northern Ireland** |
|  |  |  |  |  |  |  |
| **COVID-19 patients admitted 5^th^ February 2020 to 1^st^ August 2021** | | | | | | |
| All BMI categories | 2,721 (8,014) | 3,109 (8,632) | 3,690 (10,657) | 1,669 (5,442) | 528 (1,328) | 195 (628) |
| Underweight (<18.5 kg/m^2^) | 30 (78) | 26 (67) | 30 (85) | 10 (37) | n<5 | 2 (10) |
| Recommended (18.5-<25 kg/m^2^) | 798 (2,158) | 613 (1,561) | 740 (1,946) | 402 (1,103) | 121 (299) | 34 (114) |
| Overweight (25-<30 kg/m^2^) | 991 (2,776) | 1,083 (2,745) | 1,290 (3,413) | 506 (1,544) | 185 (418) | 60 (173) |
| Obesity 1 (30-<35 kg/m^2^) | 548 (1,664) | 703 (2,117) | 835 (2,541) | 408 (1,378) | 114 (292) | 45 (150) |
| Obesity 2 (35-<40 kg/m^2^) | 184 (734) | 382 (1,168) | 419 (1,374) | 178 (703) | 55 (170) | 30 (88) |
| Obesity 3+ (≥40 kg/m^2^) | 170 (604) | 302 (974) | 376 (1,298) | 165 (677) | 51 (146) | 24 (93) |
|  |  |  |  |  |  |  |
| **Non-COVID-19 patients admitted 1^st^ February 2018 to 31^st^ August 2019** | | | | | | |
| All BMI categories | 913 (4,577) | 1,461 (6,346) | 1,770 (7,334) | 954 (4,755) | 408 (1,481) | 131 (712) |
| Underweight (<18.5 kg/m^2^) | 91 (296) | 89 (298) | 139 (403) | 61 (235) | 15 (54) | 10 (48) |
| Recommended (18.5-<25 kg/m^2^) | 412 (1,924) | 581 (2,169) | 697 (2,628) | 399 (1,793) | 172 (550) | 48 (259) |
| Overweight (25-<30 kg/m^2^) | 247 (1,295) | 453 (1,949) | 532 (2,214) | 262 (1,371) | 136 (458) | 36 (188) |
| Obesity 1 (30-<35 kg/m^2^) | 96 (581) | 185 (1,021) | 215 (1,099) | 142 (730) | 49 (218) | 22 (114) |
| Obesity 2 (35-<40 kg/m^2^) | 38 (254) | 92 (482) | 98 (502) | 43 (329) | 24 (116) | 8 (60) |
| Obesity 3+ (≥40 kg/m^2^) | 29 (227) | 61 (427) | 89 (488) | 47 (297) | 12 (85) | 7 (43) |

Abbreviations: BMI body mass index, ICU intensive care unit
